# Cucumber mosaic virus coat protein modulates the accumulation of 2b protein and antiviral silencing that causes symptom recovery *in planta*

**DOI:** 10.1371/journal.ppat.1006522

**Published:** 2017-07-20

**Authors:** Xiao-Peng Zhang, De-Shui Liu, Teng Yan, Xiao-Dong Fang, Kai Dong, Jin Xu, Ying Wang, Jia-Lin Yu, Xian-Bing Wang

**Affiliations:** 1 State Key Laboratory of Agro-Biotechnology, College of Biological Sciences, China Agricultural University, Beijing, China; 2 College of Plant Protection, China Agricultural University, Beijing, China; University of California, Davis Genome Center, UNITED STATES

## Abstract

Shoot apical meristems (SAM) are resistant to most plant viruses due to RNA silencing, which is restrained by viral suppressors of RNA silencing (VSRs) to facilitate transient viral invasion of the SAM. In many cases chronic symptoms and long-term virus recovery occur, but the underlying mechanisms are poorly understood. Here, we found that wild-type *Cucumber mosaic virus* (CMV^WT^) invaded the SAM transiently, but was subsequently eliminated from the meristems. Unexpectedly, a CMV mutant, designated CMV^RA^ that harbors an alanine substitution in the N-terminal arginine-rich region of the coat protein (CP) persistently invaded the SAM and resulted in visible reductions in apical dominance. Notably, the CMV^WT^ virus elicited more potent antiviral silencing than CMV^RA^ in newly emerging leaves of infected plants. However, both viruses caused severe symptoms with minimal antiviral silencing effects in the *Arabidopsis* mutants lacking host RNA-DEPENDENT RNA POLYMERASE 6 (RDR6) or SUPPRESSOR OF GENE SILENCING 3 (SGS3), indicating that CMV^WT^ induced host RDR6/SGS3-dependent antiviral silencing. We also showed that reduced accumulation of the 2b protein is elicited in the CMV^WT^ infection and consequently rescues potent antiviral RNA silencing. Indeed, co-infiltration assays showed that the suppression of posttranscriptional gene silencing mediated by 2b is more severely compromised by co-expression of CP^WT^ than by CP^RA^. We further demonstrated that CP^WT^ had high RNA binding activity leading to translation inhibition in wheat germ systems, and CP^WT^ was associated with SGS3 into punctate granules *in vivo*. Thus, we propose that the RNAs bound and protected by CP^WT^ possibly serve as templates of RDR6/SGS3 complexes for siRNA amplification. Together, these findings suggest that the CMV CP acts as a central hub that modulates antiviral silencing and VSRs activity, and mediates viral self-attenuation and long-term symptom recovery.

## Author summary

In many virus-infected plants, the development of viral symptoms on the upper leaves gradually decline, until finally the top leaves appear normal and become resistant to secondary infection. Many documented cases suggest that symptom recovery is accompanied with antiviral RNA silencing. Most plant viruses encode viral suppressors of RNA silencing (VSRs) that can facilitate transient viral entry into meristems by blocking host RNA silencing defenses. However, the mechanisms of the following longer-term viral exclusion and modulation of VSRs functions remain elusive. Our studies with a substitution mutation in the CP gene demonstrate that the CMV CP has a negative role in SAM invasion. The studies suggest that during late infections, increasing CP concentrations induce potent RDR6/SGS3-dependent antiviral silencing by down-regulating accumulation of the 2b protein and induction of efficient siRNA amplification. Here, we propose a new evolutionary strategy in which the CMV CP has a role mediating viral self-attenuation and long-term symptom recovery.

## Introduction

RNA silencing is a well-established plant antiviral response triggered by viral double-stranded RNAs or highly structured single-stranded RNAs in host plants. Host Dicer-like (DCL) enzymes cleave both RNA types into 21 to 24 nucleotide (nt) small interfering RNAs (siRNAs) that are subsequently sorted into Argonaute-containing RNA-induced silencing complexes (RISC) to guide specific cleavage of the cognate viral RNAs [1–7]. Some cleavage products serve as templates for host RNA-directed RNA polymerase (RDR) 1, or RDR6, to synthesize abundant *de novo* dsRNAs that are processed by DCLs into secondary siRNAs that enhance antiviral RNA silencing [5, 7–11]. In addition, a plant-specific RNA binding protein, Suppressor of Gene Silencing 3 (SGS3), is required for siRNA amplification through forming complexes with RDR6 [12]. RNA silencing, as a major defense mechanism, occurs in all virus-infected tissues and is an extensive feature in newly emerging tissues [11, 13–15]. Recent studies have revealed that symptom recovery from viral infection is generally concomitant with induction of RNA silencing in the shoot apices of infected plants and this depends in part on RDR activity. For example, *Potato virus X* (PVX), *Turnip crinkle virus* (TCV), and *Potato spindle tuber viroid* (PSTVd) transiently invade the meristems of plant mutants defective in host *RDR6* [11, 13, 14].

As a counter-defense against host RNA silencing, many plant viruses have evolved VSRs to block various RNA silencing steps [16–18]. Some VSRs are also viral pathogenicity factors during systemic infections. For instance, the 2b protein of CMV and the 16K protein of *Tobacco rattle virus* (TRV) facilitate shoot apical meristem (SAM) invasion by blocking antiviral RNA silencing [19–21]. Ectopic expression of VSRs, like the potyvirus HC-Pro protein, enhance viral RNA accumulation of two distinct nepoviruses and prevent symptom recovery [22, 23]. Nonetheless, viral meristem invasion is transient and is followed by long-term meristem exclusion [19, 20]. Hence, the mechanisms of long-term recovery from transient SAM invasion remain to be elucidated.

CMV is the type virus of the genus *Cucumovirus* in the family *Bromoviridae*. The CMV genome is composed of three positive-stranded RNAs [24]. RNA1 and RNA2 encode 1a and 2a proteins, respectively, which comprise the viral RNA-dependent RNA polymerase subunits [24]. RNA2-derived subgenomic RNA4A encodes 2b protein, a well-known VSR and determinant factor in viral virulence and systemic infection [5, 25, 26]. RNA3 and its subgenomic RNA4 encode movement protein (MP) and coat protein (CP) that are required for viral cell-to-cell and systemic movements, respectively [24].

The CMV CP is a multifunctional factor that has roles in viral systemic movement, host range and aphid transmission [27–31]. For the Pepo and MY17 CMV strains, CP-mediated cell-to-cell movement is implicated in SAM invasion of host plants [32]. In addition, the CMV CP is an aphid transmission determinant that mediates viral spread between host plants [28, 31]. These properties and studies of different CMV strains suggest that CMV CPs are key host range determinants [30]. Previous studies have also shown that some amino acid residues of CMV CP contribute to various symptoms induction. For instance, CMV pepper strain mutants, in which proline 129 is replaced by 19 other amino acids, induce various systemic symptoms in plants [33]. Moreover, CMV CP amino acid 129 also determines viral invasion of the SAM in tobacco plants [32]. Although CMV CPs have been extensively studied as factors involved in positive regulation of viral spread and symptom induction, their negative roles in SAM infections have not been described.

In the current study, we found that the N-terminal R-rich region (R_13_RRRPRR_19_) of the CMV CP has a negative role in persistent viral SAM invasion. Our data indicate that elevated expression levels of the CMV CP induces potent antiviral RNA silencing by down-regulating the accumulation level of 2b VSRs and inducing siRNA amplification. Thus, we propose a novel self-attenuation mechanism, in which the CMV CP antagonizes the suppression effects of 2b protein and plays a pivotal role in regulating compatible interactions between CMV and host plants to prevent viral over-accumulation and persistent viral invasion of the SAM.

## Results

### The N-terminal R-rich region of CMV CP negatively regulates viral virulence in shoot apices of *N*. *benthamiana* plants

**Fig 1:**
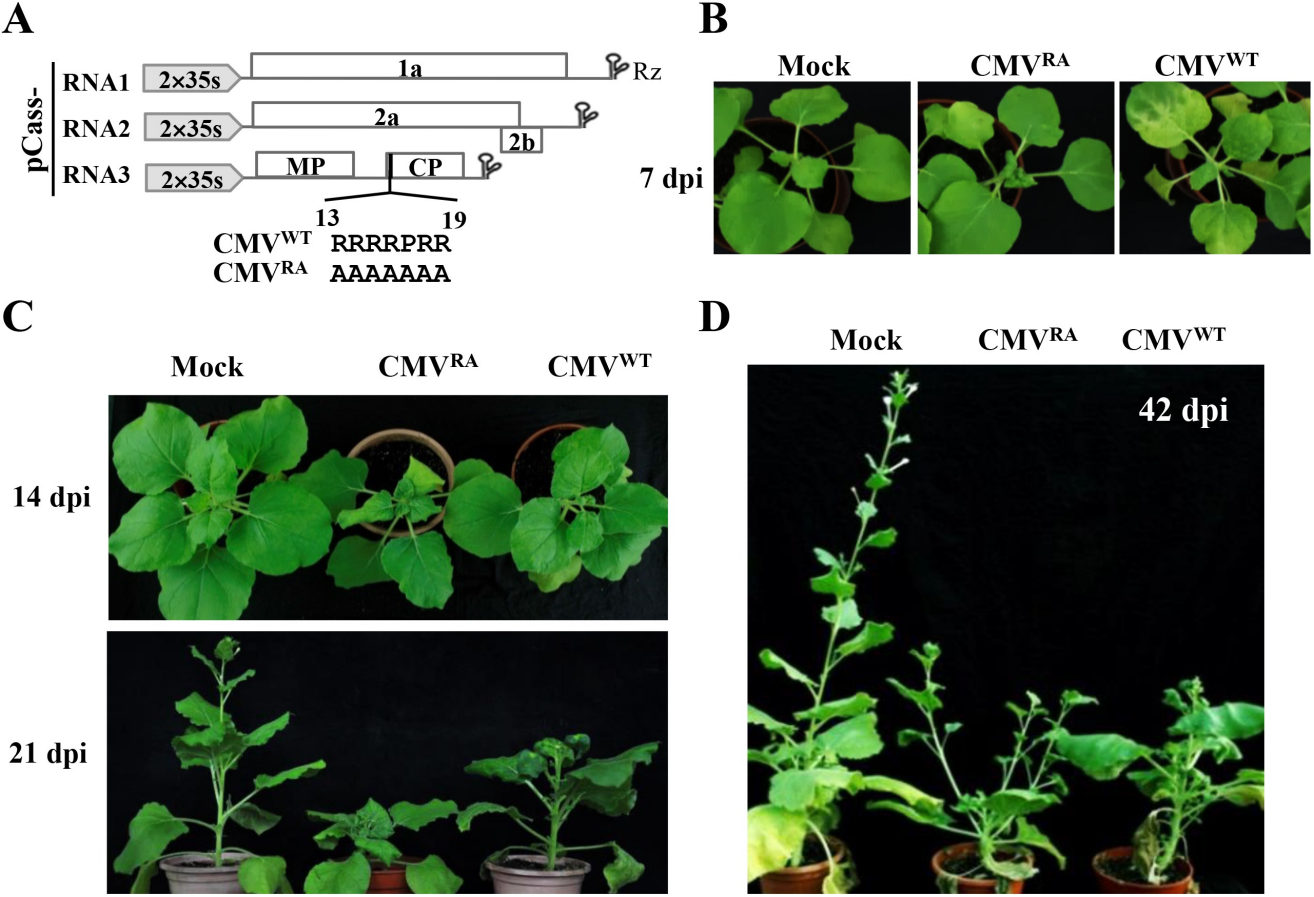
CMV CP negatively regulates viral virulence in newly emerging *Nicotiana benthamiana* leaves. (A) Schematic representations of pCass-RNA1, pCass-RNA2, and pCass-RNA3 for agroinfiltration. The full-length cDNAs of the CMV Fny strains genomic RNA1, RNA2, and RNA3 were inserted independently into pCass4-RZ between the double CaMV 35S promoter and a ribozyme sequence (Rz) derived from tobacco ringspot virus satellite RNA. The CP^WT^ and CP^RA^ harboring viruses were designated as CMV^WT^ and CMV^RA^, respectively. (B) Mild and severe symptoms elicited by CMV^WT^ and CMV^RA^, respectively, in newly emerging *N*. *benthamiana* leaves at 7 dpi. (C) Severe leaf distortion, stunting and delayed emergence of CMV^RA^ infected leaves compared with mild mosaic symptoms of CMV^WT^ at 14- and 21- dpi. Note: Compare size differences with Mock inoculated plants (D) Comparison of loss of apical dominance of CMV^RA^ versus CMV^WT^ infected plants at 42 dpi.

Plant virus-encoded CPs mainly participate in encapsidation and movement [34–38], and are increasingly appreciated as an important regulator of viral RNA replication and translation that are associated with CP RNA binding affinity [15, 39, 40]. Protein sequence analyses revealed that the N-terminal region (R_13_RRRPRR_19_) of the CMV CP is enriched in basic and positively charged amino acid residues that contribute to functional RNA binding activities. To explore the requirements of this R-rich region in viral infection, the basic amino acid residues were substituted by alanine (R13-19: A), and the resulted mutant was designated as CP^RA^ (Fig 1A). To compare the functions of CP^WT^ and CP^RA^ in the context of viral sequences, we first developed an Agrobacterium tumefaciens-mediated CMV infection system in *N*. *benthamiana* plants. The cDNAs of the three CMV genomic RNAs were engineered into pCass4-Rz to generate pCass-RNA1, -RNA2, and -RNA3 (Fig 1A). The CP^WT^- and CP^RA^-containing viruses are named CMV^WT^ and CMV^RA^, respectively. Infections were carried out by co-infiltration of equal concentrations of *A*. *tumefaciens* EHA105 mixtures harboring pCass-RNA1, -RNA2, and -RNA3 into *N*. *benthamiana* leaves.

We first explored whether CMV^RA^ could form normal viral particles during viral infections. The CMV^WT^ and CMV^RA^-infected leaves were homogenized for virions purification, and the purified virions were observed by transmission electron microscopy, which showed that CMV^WT^ and CMV^RA^ formed viral particles with similar appearance (S1A Fig). Nonetheless, the CMV^RA^ particles were less stable than CMV^WT^ virions in an RNase assay (S1B Fig). These results indicate that the CP R-rich motif is not essential for virion assembly or systemic infection in *N*. *benthamiana* plants.

The susceptibility of *N*. *benthamiana* was examined to determine the function of the R-rich region in systemic infections. At 7 dpi, CMV^WT^ induced mosaic symptoms in the fully expanded leaves, but only elicited limited or recovered symptoms in shoot apices of infected *N*. *benthamiana* plants (Fig 1B, right panel). In sharp contrast, CMV^RA^ infections resulted in severely distorted newly emerging leaves (Fig 1B, middle panel). Subsequently, CMV^RA^-infected plants exhibited extremely short internodes and petioles at shoot apices to produce a rosette appearance combined with substantial stunting between 14 and 21 dpi (Fig 1C). At 42 dpi, CMV^WT^-infected plants developed mosaic symptoms in leaves present in the central parts of the stems, and exhibited substantial reductions in growth compared to mock-infected plants (Fig 1D). However, symptoms in the shoot apices of CMV^WT^-infected plants were modulated and the apices maintained apical dominance (Fig 1D). In contrast, all of the CMV^RA^-infected plants developed several lateral shoots without distinguishable primary stems (Fig 1D). These symptoms suggested that the shoot apices were severely infected and that apical dominance was disturbed leading to production of lateral bud outgrowths [41].

Since the 2b protein is a strong silencing suppressor [5, 25, 26], we next examined whether the persistent SAM invasion by CMV^RA^ depends on the 2b protein. A 2b-deleted mutation (CMV-Δ2b) was engineered into pCass-RNA2 by point mutations as described previously [6], and co-infiltrated into *N*. *benthamiana* with pCass-RNA1 and wild-type pCass-RNA3 (CMV^WT^-Δ2b), or mutated pCass-RNA3-CP^RA^ (CMV^RA^-Δ2b). CMV^WT^-Δ2b caused mild mosaic symptoms in the systemic leaves, whereas CMV^RA^-Δ2b did not induce any obvious symptoms (S2A Fig). To explore the replication of CMV^WT^-Δ2b and CMV^RA^-Δ2b at 7 dpi, the infiltrated leaves were sampled and the CP accumulation was detected by Western blotting at 7 dpi. Both CMV^WT^-Δ2b and CMV^RA^-Δ2b CPs had accumulated to similar levels (S2B Fig), suggesting that the 2b deletion did not affect virus proliferation in infiltrated leaves. In contrast, CMV^WT^-Δ2b CP was present in the upper uninfiltrated leaves at 7 dpi, but CMV^RA^-Δ2b CP was not detected (S2C Fig). RT-PCR was performed to detect viral RNA accumulation in the upper leaves with primers corresponding to the RNA3 CP region. CP specific bands were detected in plants inoculated with CMV^WT^-Δ2b, but not with CMV^RA^-Δ2b (S2D Fig). Collectively, these data demonstrate that CMV CP^WT^ exerts a negative role in shoot apex infections, and 2b protein is required for systemic infection of the CMV^RA^ mutant.

### CMV^RA^, but not CMV^WT^, persistently invades the shoot apices of infected plants

**Fig 2:**
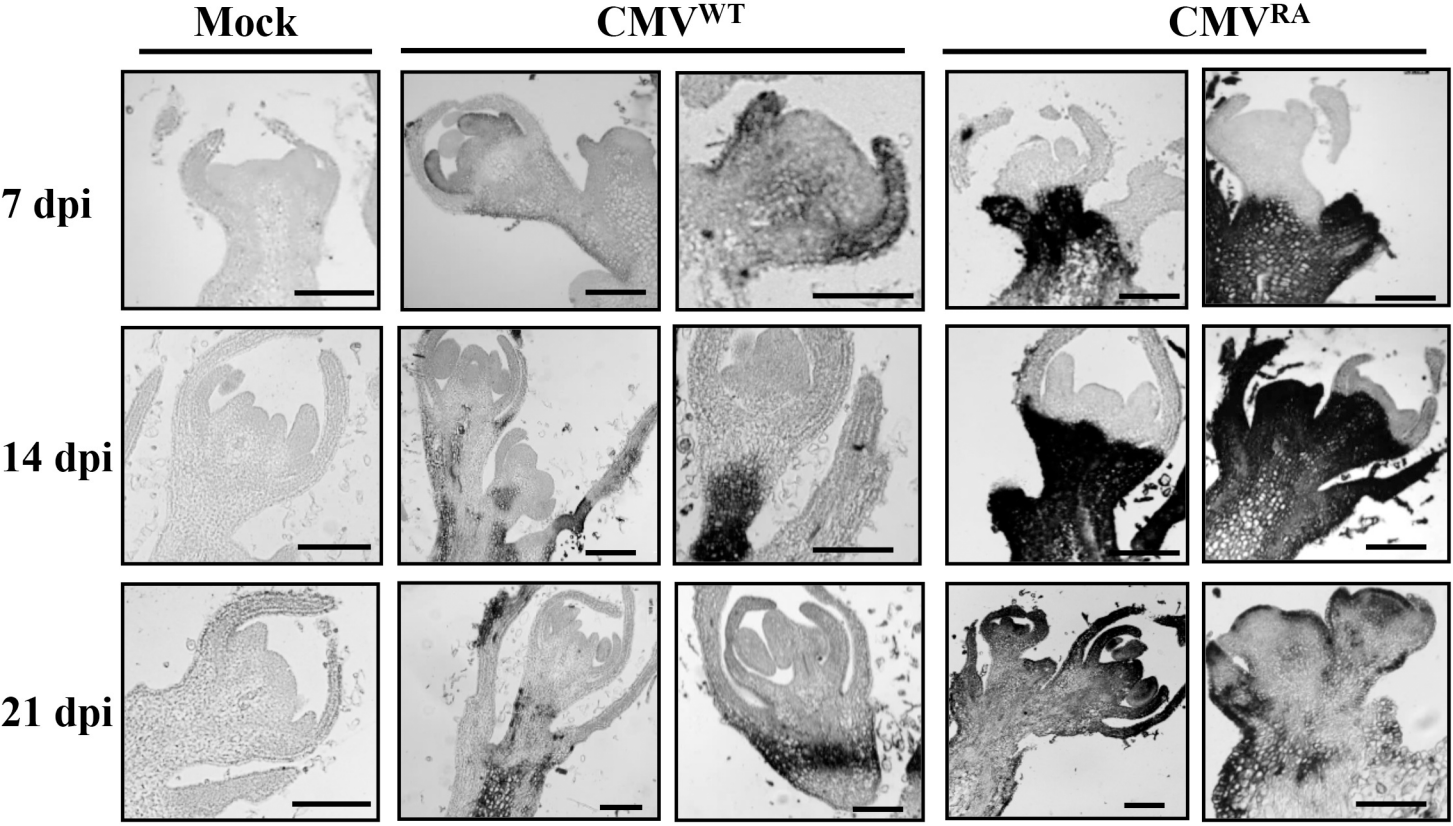
Distribution of CMV^RA^ or CMV^WT^ in the apical meristems of infected plants. *In situ* hybridization of longitudinal sections of shoot apices in *N*. *benthamiana* plants inoculated with CMV^RA^ and CMV^WT^ at 7-, 14-, and 21- dpi. Dark areas indicate the presence of viral RNA revealed by digoxigenin-labeled riboprobe corresponding to the CMV CP. CMV signals were not observed in the shoot meristem from uninfected samples (left panel). Only a low signal density was present in CMV^WT^ infected meristems at 7 dpi and these disappeared from the meristem by 14 dpi (middle panels). In contrast, CMV^RA^ infection contained abundant signal densities beneath the meristems at 7 dpi, and indicated partially invaded meristems by 14 dpi, and completely invaded meristems at 21 dpi (right panels). Bars = 100 μm.

Because apical dominance was abolished in CMV^RA^ infections, we proposed that CMV^RA^ invaded meristems and altered the meristematic activity of the infected plants. To explore this possibility, longitudinal sections of the topmost flowers and shoot apices from mock-, CMV^WT^-, and CMV^RA^-infected plants were examined by *in situ* hybridization with digoxigenin-labeled CMV RNA3 probes. CMV^WT^ invaded most shoot meristems and floral primordia by 7 dpi, but subsequently disappeared between 14 and 21 dpi, despite of some detectable signals below the SAM and floral primordia (Fig 2, middle panels). In contrast, CMV^RA^ was abundant below the meristems at 7 dpi, and partially moved into the meristems by 14 dpi (Fig 2, right panels). CMV^RA^ invaded the meristems of all the infected plants by 21 dpi (Fig 2, right panels), but as expected were absence in meristems from mock inoculated plants (Fig 2, left panels). These results demonstrate that CMV^RA^ accumulated to high levels and resulted in severe stunting and abolished apical dominance, whereas CMV^WT^-infected plants recovered from meristem infection.

### CMV^WT^ induces more potent antiviral silencing than CMV^RA^ in emerging *N*. *benthamiana* tissues

**Fig 3:**
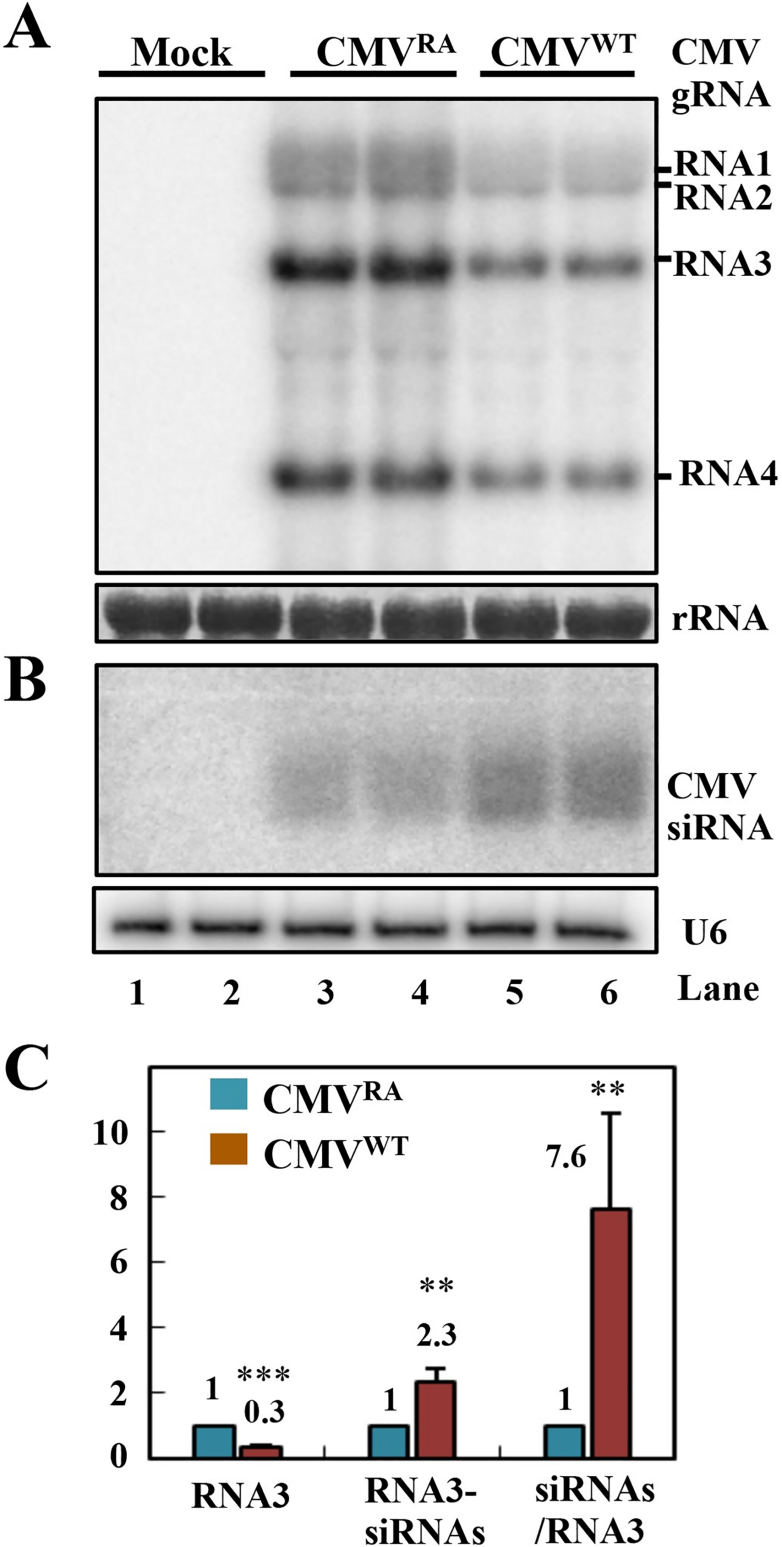
CMV^WT^ induces more potent antiviral silencing than CMV^RA^ in emerging *N*. *benthamiana* leaves. (A) Accumulation of CMV gRNAs and sgRNAs and (B) RNA3-vsiRNAs in emerging *N*. *benthamiana* leaves at 7 dpi with CMV^RA^ and CMV^WT^. Loading controls for the high and low molecular weight RNAs were rRNA and U6 RNAs, respectively. (C) Ratios of accumulation levels of CMV gRNA3 and RNA3-vsiRNAs calculated from signal intensities in three independent hybridization experiments. The vsiRNAs/RNA3 values refer to the relative ratios of RNA3-vsiRNAs versus viral genomic RNA3. The values of RNA3 and RNA3-vsiRNAs in CMV^RA^-infected leaves were set as 1. ** *P*-value < 0.01; *** *P*-value < 0.001.

Because RNA silencing is a key antiviral mechanism in meristems infections [11, 13, 14, 19–21], we analyzed levels of viral RNA and siRNAs by Northern blotting at 7 dpi. The newly grown tissues were collected for the Northern blotting analysis of viral RNA and siRNA at 7 dpi. A markedly increased accumulation of viral RNA of CMV^RA^ was detected in the newly grown tissues compared with those of CMV^WT^ (Fig 3A, compare lanes 3, 4 with 5, 6). However, CMV^RA^ RNA3-derived siRNAs (RNA3-vsiRNAs) were present at a much lower level than those of CMV^WT^ (Fig 3B, compare lanes 3, 4 with 5, 6). To determine the relative accumulation levels of viral genomic RNA3 and RNA3-vsiRNAs, the hybridization signal intensities in three independent experiments were evaluated. The values of RNA3 and RNA3-vsiRNAs in CMV^RA^-infected plants were set as one unit. CMV^WT^ RNA3 accumulated to about 30% of CMV^RA^ RNA3 (Fig 3C, left panel, *P*-value < 0.001). However, the levels of CMV^WT^ RNA3-vsiRNAs were 2.3 times those of CMV^RA^ RNA3-vsiRNAs (Fig 3C, middle panel, *P*-value < 0.01). The relative RNA3-vsiRNAs/RNA3 ratio in the CMV^WT^-infected newly emerging tissues was six-fold higher than that of CMV^RA^-infected plants (Fig 3C, right panel, *P*-value < 0.01), indicating that CMV^WT^ induces more potent antiviral silencing than CMV^RA^ in the emerging tissues.

Collectively, CMV^WT^ promotes the accumulation of vsiRNAs that inhibit virulence in the shoot apices of infected plants. In contrast, the lower amounts of CMV^RA^ vsiRNAs reduced antiviral silencing and enabled CMV^RA^ to persistently infect the newly emerging tissues.

### SGS3 and RDR6 are required for potent antiviral RNA silencing induction by CMV^WT^ in emerging tissues of infected *Arabidopsis thaliana*

**Fig 4:**
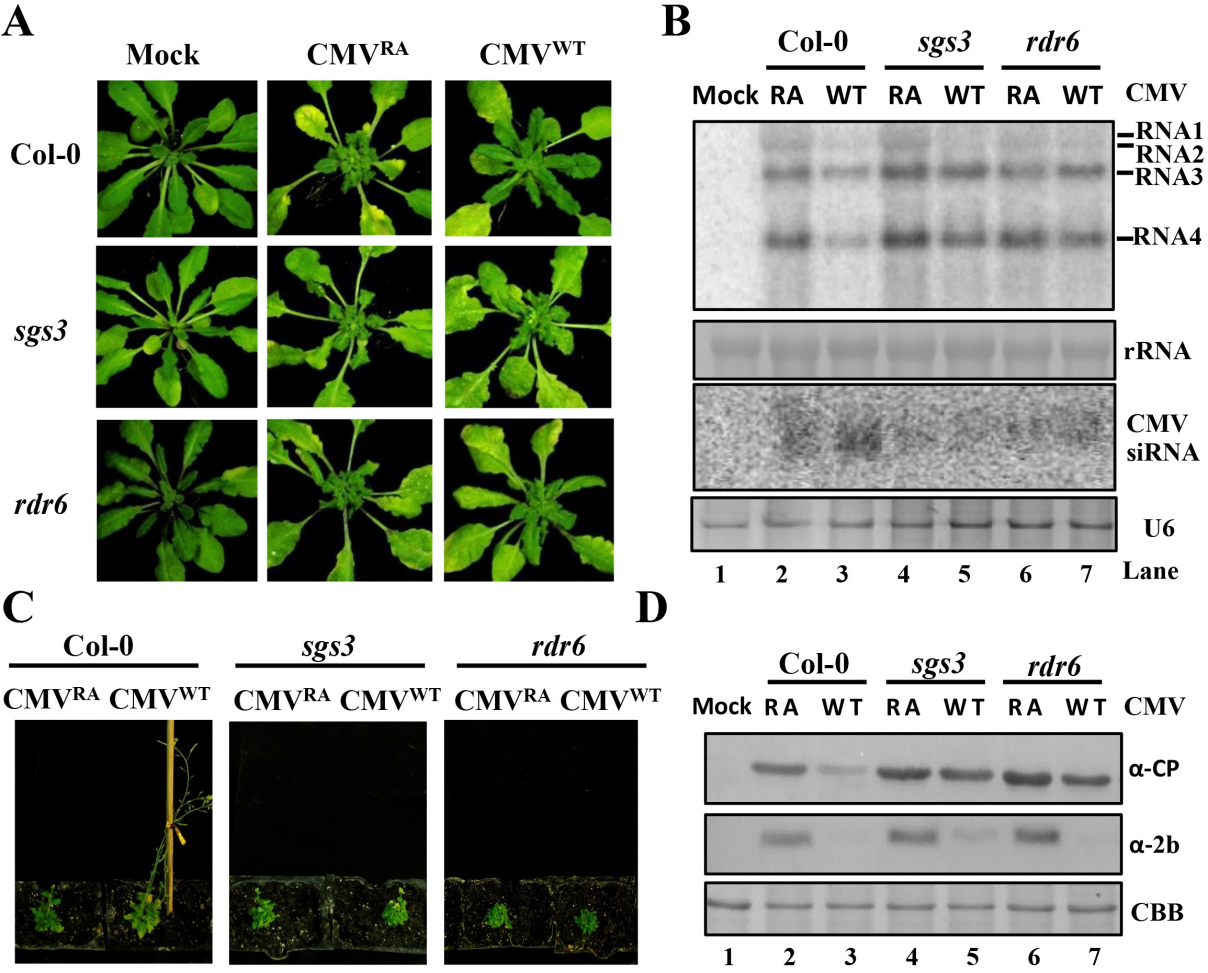
CMV^WT^ induced more potent RDR6/SGS3-dependent antiviral silencing than CMV^RA^ in newly emerging tissues of infected *Arabidopsis thaliana*. (A) CMV^WT^ and CMV^RA^ symptoms in newly emerging tissues of Col-0, *sgs3*, and *rdr6* mutant plants at 21 dpi. CMV^WT^ caused severe symptoms in emerging tissues of *sgs3* and *rdr6* mutant plants, but not in those of Col-0 plants. In contrast, CMV^RA^ caused similar severe symptoms in Col-0, *sgs3*, and *rdr6* plants. (B) Accumulation of CMV gRNAs/sgRNAs and RNA3-vsiRNAs in emerging leaves of *A*. *thaliana* plants infected with CMV^RA^ or CMV^WT^ at 21 dpi. Loading controls for the high and low molecular weight RNAs were rRNA and U6 RNAs, respectively. (C) Comparison of CMV^WT^ and CMV^RA^ symptoms Col-0, *sgs3* and *rdr6* plants at 56 dpi. CMV^WT^ elicited mild mosaic and lack of stunting in Col-0 plants, whereas CMV^RA^ plants were severely stunted, had reduced apical dominance and bolted much later than CMV^WT^ plants. Both *sgs3* and *rdr6* plants developed severe symptoms after infection with CMV^RA^ and CMV^WT^. (D) Accumulation of CMV CP and 2b protein in the *A*. *thaliana* emerging leaves shown in panel A. Anti-CP, and -2b polyclonal antibody were used to detect the accumulation of CP and 2b protein accumulation, respectively. Coomassie brilliant blue (CBB) staining was used as the protein loading control.

To provide more detailed genetic analyses of host effects on CMV infection, we investigated the impacts of the CMV CP derivatives during infection of *Arabidopsis thaliana*. CMV^WT^ induced visible disease symptoms in the fully expanded leaves of Columbia-0 (Col-0) wild-type plants at 21 dpi (Fig 4A and S3A Fig). These plants had similar bolting times as the mock inoculated plants and maintained apical dominance during late infection stages between 35- and 56- dpi (S3A Fig). Compared with CMV^WT^, CMV^RA^ infection resulted increased numbers of distorted leaves in the newly emerging tissues (Fig 4A and S3A Fig), late bolting, and reduced apical dominance in the late infection stages (S3A Fig). Northern blotting results of viral RNA and vsiRNAs extracted from CMV^WT^- and CMV^RA^-infected systemic leaves at 21 dpi were consistent with those of infected *N*. *benthamiana*, showing that CMV^RA^ infected plants accumulates higher levels of viral genomic RNA and less vsiRNAs compared with CMV^WT^ infections in both plant species (Fig 4B and S3 Fig). Thus, CMV^WT^ elicits more potent antiviral silencing than CMV^RA^ in emerging tissues of infected *A*. *thaliana*, and is similar in this regard to infected *N*. *benthamiana* plants.

To explore whether CMV^WT^ induction of potent antiviral silencing depends on host RDRs, we inoculated *A*. *thaliana rdr6* and *sgs3* mutants with CMV^WT^ or CMV^RA^. The *rdr6* and *sgs3* mutants infected with CMV^RA^ displayed severe symptoms similar to those of wild-type plants and whole plant development was restrained (Fig 4A and Fig 4C). The *rdr6* and *sgs3* mutant plants infected with CMV^WT^ exhibited more severe disease symptoms in the newly emerging tissues compared with wild-type plants (Fig 4A and Fig 4C). In addition, Northern RNA hybridizations revealed that the accumulation level of viral RNA in CMV^WT^-infected wild-type plants was dramatically lower than those of infected *rdr6* or *sgs3* plants, whereas higher levels of vsiRNAs accumulated in infected wild-type plants than in *rdr6* or *sgs3* plants (Fig 4B, compare lanes 3, 5, and 7). In contrast, high levels of viral RNA and low levels of vsiRNAs were detected in the emerging tissues of Col-0, *rdr6* and *sgs3* mutants infected with CMV^RA^ (Fig 4B, compare lanes 2, 4, and 6). Collectively, these results demonstrate that RDR6 and SGS3-dependent amplification of vsiRNAs is required for the CMV^WT^-induced potent antiviral RNA silencing in emerging tissues of CMV^WT^ infected plants.

We next investigated the accumulation of the CP and 2b protein in emerging tissues of CMV infected Col-0, *rdr6*, and *sgs3* mutants. Accumulation of CMV^WT^ CP in the *rdr6* and *sgs3* mutants increased to level similar to those of CMV^RA^ CP (Fig 4D, top panel). Notably, accumulation of CMV^WT^ 2b protein was lower level in the *rdr6* and *sgs3* mutants compared with CMV^RA^ infection, indicating that the accumulation of 2b protein was significantly down-regulated in CMV^WT^-infected plants (Fig 4D, middle panel). Collectively, these results demonstrate that the down-regulated 2b protein cannot efficiently inhibit RDR6/SGS3-dependent antiviral silencing that restricts elevated accumulation of CMV^WT^ in emerging tissues.

### CMV CP attenuates 2b-mediated suppression of local GFP silencing in a dose-dependent manner

**Fig 5:**
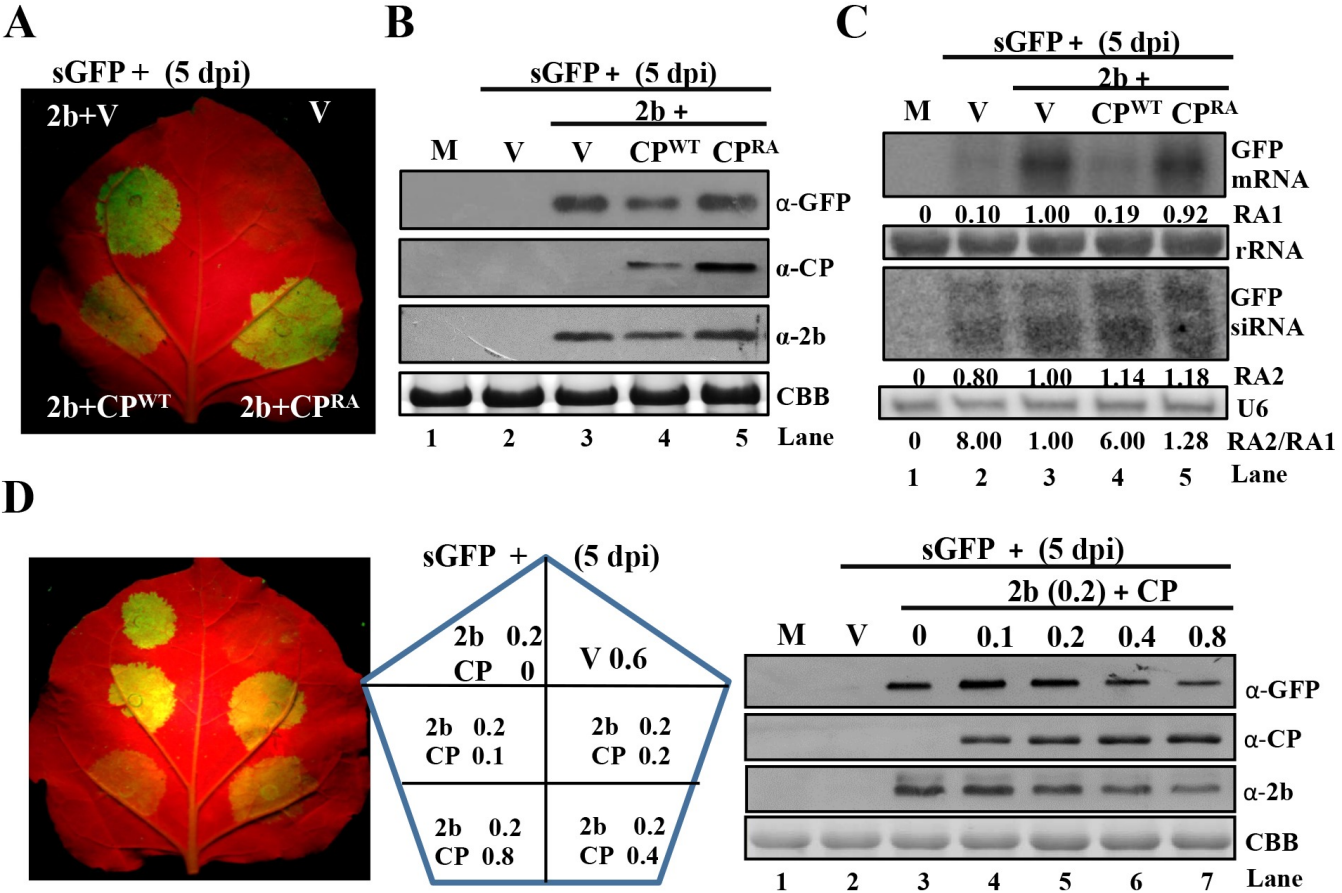
CMV CP attenuates 2b-mediated suppression of local GFP silencing. (A) GFP fluorescence in regions of *N*. *benthamiana* leaves after agroinfiltration of sGFP reporter gene (OD_600_ = 0.4), in combination with different amounts of the pGD empty vector (V, OD_600_ = 0.4), the pGD-2b (OD_600_ = 0.2) and the pGD-CP vectors (OD_600_ = 0.4), as indicated. Photographs were taken under UV light at 5 dpi. (B) Protein gel blot analysis of samples extracted from infiltrated region shown in panel A. (C) Accumulation of GFP mRNA and siRNAs in the region shown in panel A. Methylene blue-stained rRNA and U6 RNA were used as loading controls for high and low molecular weight RNAs, respectively. The values under GFP mRNA (RA1) and siRNAs (RA2) represent the relative accumulation (RA) of GFP mRNA and GFP-derived siRNAs, respectively. The RA values of sGFP with 2b and V were set as 1. RA2/RA1 ratios under U6 detection represent the relative production of GFP-derived siRNAs versus GFP mRNA. (D) GFP fluorescence (left panel) in local patches agroinfiltrated with the sGFP vector (OD_600_ = 0.4), empty pGD vector, and the 2b vectors (OD_600_ = 0.2), either alone or in combination with CP^WT^ or CP^RA^ vectors (OD_600_ = 0–0.8), as indicated in middle panel. A protein gel blot analysis of samples extracted from the infiltrated regions is shown in the right panel. Anti-GFP, -CP, and -2b polyclonal antibodies were used to assess the GFP, CP, and 2b, accumulations, respectively. Mock-infected plants were used as the negative control. Coomassie brilliant blue (CBB) staining was used as the protein loading control.

Our findings have demonstrated that CP^WT^ exerts negative effects during viral SAMs infection. To independently verify the antagonistic roles of CP and 2b, we next examined the effects of CP^WT^ and CP^RA^ on VSR activities of 2b by co-infiltration assays in *N*. *benthamiana* plants [42]. Green fluorescence occurring early during transient co-expression of GFP disappeared completely at 5 dpi, indicating that potent silencing was induced (Fig 5A). In contrast, high intensity of GFP fluorescence in the regions of leaves co-expressing 2b and GFP suggested that GFP silencing was efficiently suppressed by the 2b protein (Fig 5A). We next compared local GFP silencing suppression by 2b co-expressed with the empty vector (V), CP^WT^, or CP^RA^, respectively. The CP^WT^, unlike V or CP^RA^, significantly attenuated the 2b-mediated suppression of GFP silencing (Fig 5A). The observations were further verified by Western blotting showing that co-expression of CP^WT^ markedly reduced the expression levels of GFP protein expression compared to co-expression of V and CP^RA ^(Fig 5B, top panel). Simultaneously, the CP and 2b protein also accumulated to lower level during co-expression with CP^WT^ than co-expression with V and CP^RA^ (Fig 5B, middle and bottom panels).

To examine the silencing potency in infiltrated regions of *N*. *benthamiana* leaves, the accumulation of GFP mRNA and siRNAs was compared by Northern blotting analyses, and hybridization signal densities were measured to determine the relative accumulation of mRNA and siRNAs. The values from leaf samples co-expressing GFP, 2b and V were set as one unit. The GFP mRNA expression level in the sample co-expressing 2b protein and CP^WT^ significantly decreased compared with those of V or CP^RA^ (Fig 5C, top panel and RA1 values, compare lane 4 with lanes 3 and 5), but all patches accumulated similar levels of GFP-derived siRNAs (Fig 5C, middle panel and RA2 values, compare lane 4 with lanes 3 and 5). The relative accumulated levels of GFP siRNAs versus GFP mRNA (RA2/RA1) were compared. The GFP siRNAs/mRNA ratios in the patches co-expressing 2b and CP^WT^ were at least four-fold higher than those co-expressing 2b with V or CP^RA^ (Fig 5C, RA2/RA1 values, compare lane 4 with lanes 3 and 5), demonstrating that CP^WT^ enhances the potency of GFP silencing.

During CMV infection, the accumulation of the CP increases gradually during viral propagation. Therefore, we wondered whether CP affected VSR activities of 2b in a dose-dependent manner. To answer this question, we compared the GFP fluorescence from the different patches infiltrated with a CP concentration gradient (OD_600_ = 0, 0.1, 0.2, 0.4, and 0.8) and 2b (OD_600_ = 0.2) (Fig 5D, middle panel). A low concentration of infiltrated CP (OD_600_ = 0.1) had negligible effects on 2b-mediated suppression of GFP silencing, whereas increasing CP concentrations gradually compromised the inhibitory effects as the increasing concentration of CP, and the highest CP concentration (OD_600_ = 0.8) significantly down-regulated the 2b suppression (Fig 5D, left panel). Additionally, Western blotting analyses revealed that the accumulation of GFP and 2b decreased in proportion to the increasing concentrations of co-expressed CP (Fig 5D, right panel). Thus, our results indicate that low CMV CP levels do not affect 2b suppressive activities, whereas highly abundant CMV CP concentrations down-regulate 2b protein accumulation and attenuate 2b-mediated silencing suppression. The R-rich region of CP is highly conserved in many subgroup I and subgroup II CMV strains, as well as in *Tomato aspermy virus* (TAV) (S4A Fig), and all the CPs exert negative effects on 2b-mediated suppression of RNA silencing (S4 Fig).

In the field, synergistic viral diseases are usually caused by interactions of different viruses that result in dramatically increased viruses titer and symptom induction, which are mainly dependent on VSRs activities [43]. With regard to the co-infection of CMV with other plant viruses in the field, we postulated that CMV CP compromises the suppression activity of other VSRs in co-infected plants. To test this hypothesis, we co-infiltrated CP with P19, P38, or HC-Pro in *N*. *benthamiana* leaves. In agreement with 2b, the suppressions mediated by the VSRs were attenuated by coinfiltration with CP^WT^, but this effect was not observed with the CP^RA^ (S5A Fig). The results were also confirmed by Western blotting which revealed a substantial reduction of GFP accumulation in the patches co-expressing of VSRs and CP^WT^ compared with VSRs and CP^RA^ (S5B Fig). At the same time, the co-expression of CP^WT^ rather than CP^RA^ also decreased the accumulation of the VSRs (S5B Fig). We further demonstrated that the compromising effect of the CMV CP on P19-mediated suppression was also dependent on CP^WT^ concentrations (S5C Fig). In conclusion, highly abundant CMV CP concentrations compromise various VSRs suppression activities in patch assays, implying that the CMV CP modulates the synergistic viral disease by regulating silencing interactions and VSRs in the co-infected plants.

### CMV CP^WT^ has strong RNA-binding affinity and mediates translation inhibition

**Fig 6:**
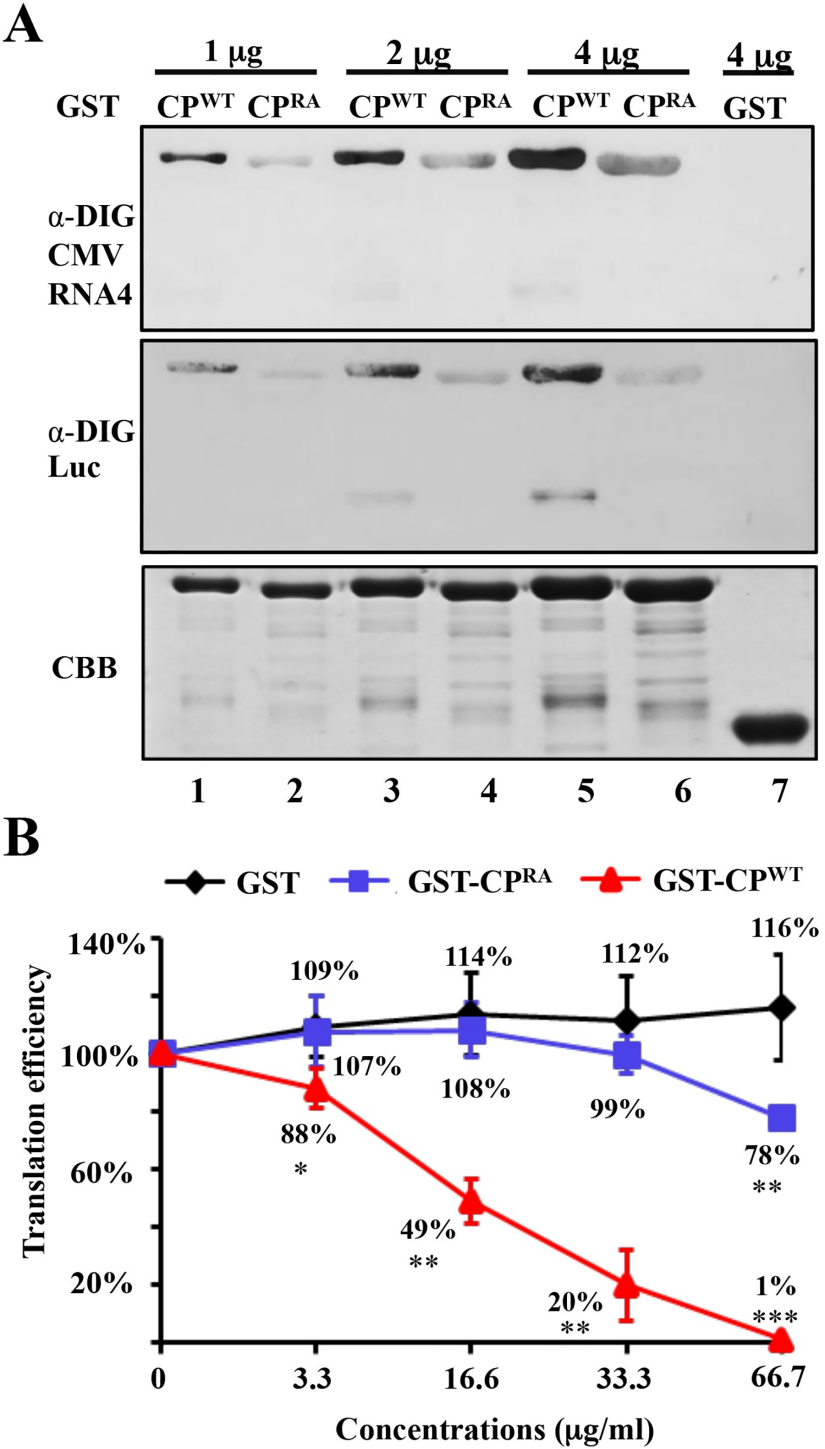
RNA binding and translation inhibition by the CP^WT^ and CP^RA^ proteins. (A) RNA-binding abilities of the GST-CP^WT^ and GST-CP^RA^ proteins as assessed by digoxigenin-labeled CMV RNA4 or Luciferase (Luc) mRNA (See Materials and methods for details). GST served as a negative control. Coomassie brilliant blue (CBB) staining was used as the protein loading control. (B) Translation inhibition of Luciferase mRNA by CP^WT^ and CP^RA^ *in vitro*. The Luc mRNA was transcribed and incubated with different concentrations of the GST, GST-CP^RA^, or GST-CP^WT^ in wheat germ extract at 25°C for two hours. Then, the activity of translated Luciferase *in vitro* was measured with a luminometer. Luciferase activities from mRNA without GST, GST-CP^RA^, or GST-CP^WT^ was set as 100%. Error bars represent the standard error of the mean. Data points are the mean value of three independent experiments. *P-value < 0.05; ** *P*-value < 0.01; *** *P*-value < 0.001.

The decreased accumulation of VSRs by high-abundant CMV CP might be due to the strong RNA binding activity of CP^WT^ and resulting in translation inhibition. To test this possibility, we carried out North-Western blotting assays to compare the RNA binding affinity of CP^WT^ and CP^RA^. GST-tagged CP^WT^, CP^RA^, and untagged GST were expressed and purified from *E*.*coli* expression systems. Different amounts (1 μg, 2 μg, and 4 μg) of GST-CP^WT^ and GST-CP^RA^ were separated in SDS-PAGE gels, transferred to nitrocellulose membranes, renatured, and exposed to digoxigenin-labeled CMV RNA4 or luciferase (Luc) mRNA. High amounts (4 μg) of GST served as a negative control and failed to binding the RNAs (Fig 6A, lane 7), confirming that the GST tag does not have RNA-binding affinity. The GST-CP^WT^ bound much higher levels of CMV RNA4 and Luc mRNA than the GST-CP^RA^ protein (Fig 6A, compare lanes 2, 4 and 6 with lanes 1, 3 and 5). Together, these results clearly indicate that the R-rich region is important for the high RNA binding affinity of CMV CP^WT^.

We further examined whether the high unspecific RNA binding activity of CP^WT^ resulted in translation inhibition by comparing *in vitro* translation efficiency in the wheat germ system. The results showed that high concentrations of GST tag did not affect the translation of Luc mRNA (Fig 6B, black line). The highest concentration (66.7 μg/ml) of GST-CP^RA^ had a~ 20% translation reduction compared with the empty control (*P*-value < 0.01) (Fig 6B, blue line), indicating that the weak RNA binding of the GST-CP^RA^ protein partially affected mRNA translation. However, more dramatic reductions in the luciferase translation effects were observed as the GST-CP^WT^ concentration was gradually increased, and finally the highest concentration (66.7 μg/ml), was ~1% of the empty control (*P-*value < 0.001) (Fig 6B, red line). These results are in agreement with previous studies, in which the *Potato virus A* (PVA) CP was found to be involved in inhibition of viral RNA translation [44, 45]. Therefore, it appears that the strong RNA-binding affinity and resulting translation inhibition by the CMV CP^WT^ contributes to reduced VSRs accumulation in CMV^WT^ virus infections.

### CMV CP associates with SGS3 and RDR6 protein in punctate granules *in vivo*

In previous studies, high CP concentrations of several positive-sense RNA viruses repressed RNA translation and facilitated virion assembly [44, 46, 47]. Similarly, our results show that CMV CP also efficiently inhibits translation of Luc mRNA in wheat germ system (Fig 6B). In addition to initiating viral encapsidation and/or CMV ribonucleoprotein (RNP) formation, translation inhibition also contributes to elevated accumulation of aborted mRNA transcripts without translation that might be recognized as aberrant RNA by host RDR to initiate siRNAs amplification. SGS3, acting as co-factor of RDR6, mainly binds to and stabilizes RNA substrates to amplify secondary siRNAs [48–50].

**Fig 7:**
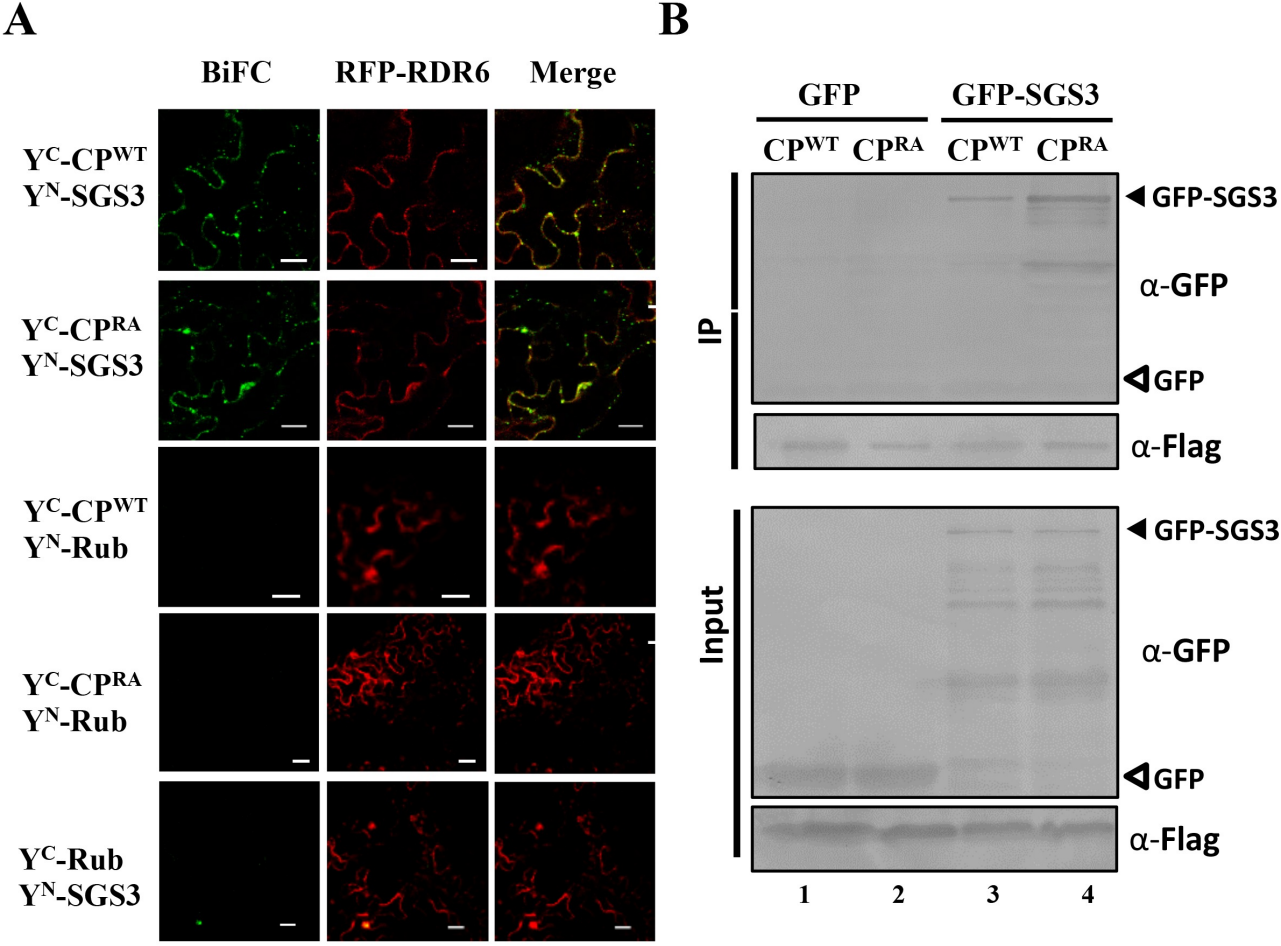
CMV CP is associated with RDR6/SGS3 complex. (A) Interactions detection of SGS3 with CP^WT^ or CP^RA^ in *N*. *benthamiana* leaves epidermal cells using bimolecular fluorescence complementation (BiFC). Leaves were infiltrated with pairs of Agrobacterium strains harboring tested proteins tagged with the different halves of YFP. Rubisco protein (Rub) was negative control. Images were taken at 2 dpi by confocal scanning laser microscopy. Bars represent 20 μm. (B) Co-immunoprecipitation (Co-IP) assays showing associations of SGS3 with CPs *in vivo*. *N*. *benthamiana* leaves coexpressing Flag-CP^WT^ and Flag-CP^RA^ proteins in combination with the GFP-SGS3 protein were homogenized and incubated with anti-Flag M2 antibodies for co-IP assays. GFP was included as a negative control. Anti-GFP and anti-Flag antibodies were used to detect accumulations of GFP-SGS3/GFP and CP, respectively. The positions of GFP-SGS3 and GFP are indicated by black and white triangles, respectively.

To visualize potential CP–RDR6/SGS3 protein associations in living cells and their subcellular occurrence, we conducted bimolecular fluorescence complementation (BiFC) assays with Agrobacterial-infiltrated leaves of *N*. *benthamiana*. For this assay, the SGS3 and RuBisco proteins (Rub) were fused with the N-terminal half of sYFP, and the tagged CP^WT^, CP^RA^ and Rub proteins were fused with the C-terminal half of sYFP. YFP^C^-CP^WT^ and YFP^C^-CP^RA^ could be associated with YFP^N^-SGS3 in the cytoplasm, and formed punctate granules that co-localized with RFP-tagged RDR6 (Fig 7A, top two panels). BiFC fluorescence was not detected in the Rub control samples that were co-expressed with CP or SGS3 (Fig 7A, three bottom panels). To further evaluate the CP–SGS3 protein association *in vivo*, we performed co-immunoprecipitation (co-IP) assays *in planta*. In these experiments, Flag-CP^WT^, Flag-CP^RA^ proteins were co-expressed with GFP-SGS3 or GFP proteins, and extracts from infiltrated leaves were used in co-IP assays with anti-Flag beads. Both the Flag-CP^WT^ and Flag-CP^RA^, immunoprecipitated GFP-SGS3 efficiently, but the GFP protein did not (Fig 7B). Nevertheless, we could not detect the interaction of CPs and SGS3 through a yeast-two-hybrid assay (S6 Fig), hence the association of CPs and SGS3 in *N*. *benthamiana* might be indirect. Collectively, both CP^WT^ and CP^RA^ appear to colocalize with SGS3 in punctate granules *in vivo* and RDR6 also is present in these granules. However, in contrast with CP^RA^, the CP^WT^ protein has a high affinity with RNA to result in general inhibition of host RNA and viral RNA translation. This inhibition results in production of aberrant RNAs that in turn appear to be associated with SGS3 and RDR6 for siRNA amplification.

In summary, we conclude that the CMV CP^WT^ protein RNA binding contributes indirectly to high potency RNA silencing that presumably results in reduced accumulation of the CMV 2b protein. Reductions in 2b VSRs activities in turn result in a cycle in which increased siRNAs production by RDR6/SGS3 dependent amplification leads to reductions in CMV RNAs, elevated virus attenuation, and protracted symptom recovery in newly emerging leaves of infected plants.

## Discussion

Symptom recovery represents an extreme virus attenuation effect, in which, infected plants initially develop sever leaf symptoms, but subsequently newly emerging leaves exhibit a drastically reduced virus accumulation due to induction of antiviral RNA silencing [15, 51, 52]. In previous studies, host RNA silencing effects and interactions with the CMV Pepo strain 2b protein were shown to be involved in transient appearance of CMV in meristems at 7 dpi, and decreases in virus concentration as new leaves emerged and disappearance in recovered tissues [19, 21]. In agreement with these results, we found that CMV Fny strain also infected SAM transiently and then was excluded from the shoot apices leading to symptom recovery (Fig 1 and Fig 4). Our studies unexpectedly found that a mutant harboring an alanine substitution in the N-terminal R-rich region of the CMV CP could persistently invade meristems and block the growth of apical shoots, implying that CMV CP^WT^ facilitates long-term SAM exclusion at late infection stages (Fig 1 and Fig 2). The previous studies have shown that the amino acid 129 of the Pepo CMV CP affects the cell-to-cell movement and determines successful SAM invasion in tobacco plants at 6–8 dpi, but the shoot meristems recovered from the Pepo infection at 21 dpi [21, 32]. Combined with previous studies, our results indicate that CMV CP is not only required for successful SAM invasion at the early infection, but also modulates viral exclusion from SAM later in infection. Thus, we propose that the CMV CP plays a critical role in compatible interactions between CMV and host plants.

Plant viral CPs have a primary function involving viral genome encapsidation, but also have been implicated in viral translation and/or replication. At low concentrations, viral CPs usually facilitate RNA replication and/or translation, whereas at higher concentrations, they may inhibit these processes in favor of virion assembly [39, 40, 44–46]. For instance, the *Brome mosaic virus* (BMV) CP binds to an RNA element within the 5´UTRs of the viral genome and suppresses the translation of RNA replication proteins [46]. In addition, the *Hepatitis C virus* (HCV) core protein binds specifically to the internal ribosome entry site (IRES) in the 5´UTR of the viral genome [47, 53–55]. This binding requires positively-charged residues in the N-terminal portion of the core protein and results in suppression of translation of downstream genes. In addition, lysine-to-alanine mutations in the N-terminal region of *Red clover necrotic mosaic dianthovirus* (RCNMV) CP induced more severe symptoms than wild-type virus in *N*.*benthamiana*, indicating that the lysine-rich N-terminus of RCNMV CP modulates symptomatology, independently of its role in virion assembly [56]. In line with these examples, we have shown that the CMV CP has high unspecific RNA binding activities and inhibits the translation of Luc mRNA protein in the wheat germ system (Fig 7). The mutant CP^RA^ harboring an alanine substitution in the N-terminal R-rich region was significantly compromised in translation inhibition, implying that the basic and positively charged amino acid residues are required for translation repression by the CMV CP (Fig 7). The high concentrations of CMV CP in the wheat germ system resemble CP concentrations at late stages of infection when the CP is among the most prominent proteins in the cell. Additional experiments are required to explore the binding regions of CMV CP in the mRNA and the viral genomic/subgenomic RNA interactions. Given the well-known functions of 2b as viral virulence determinants and silencing suppression [6, 9, 26, 57–63], we postulate that decreased accumulation of 2b protein by saturated CP directly or indirectly contributes to potent antiviral RNA silencing that resulting in virus exclusion from SAM and symptom recovery. However, we cannot exclude that other host and/or viral components possibly affected by the CP are involved in symptom recovery. For example, non-specifically binding of RNAs by the CP^WT^ protein might disturb metabolic processes of the host and lead to low virus accumulation in the SAM. Another possibility is that unstable particles assembled by the CP^RA^ mutant permit re-infections in the same cells and therefore lead to higher RNA accumulation. Future studies are anticipated to provide exciting insights into symptom recovery processes.

In the process of RNA silencing, host RDRs are required to initiate or amplify RNA silencing via dsRNA synthesis, and the substrates for dsRNA synthesis *in vivo* are aberrant RNA lacking a cap structure or poly(A) tails. Arabidopsis *ETHYLENE-INSENSITIVE5 (EIN5)*/ EXORIBONUCLEASE4 (XRN4) encodes a cytoplasmic 5′-3′ exoribonuclease that degrades RNA intermediates derived during mRNA decay and/or RISC slicing, and regulates RDR6-dependent production of siRNAs [64, 65]. Arabidopsis *Super-Killer2* (SKI2) functions as a cytoplasmic SKI complex to unwind RNAs into the 3´to 5´exoribonuclease complex for decay, and acts as a repressor of endogenous PTGS [66]. Therefore, both 5´to 3´and 3´to 5´cytoplasmic RNA decay pathways function in RDR-dependent silencing. Here, we propose that the high binding activity of CP^WT^ with RNA protects viral RNA intermediates from RNA decay, which increases the substrate concentration of RDR/SGS3 complex and subsequently improves host antiviral silencing. We consistently found that CMV^WT^-induced antiviral silencing is significantly compromised in emerging leaves of *sgs3* and *rdr6* Arabidopsis mutants compared with those of wild-type Arabidopsis plants (Fig 4).

**Fig 8:**
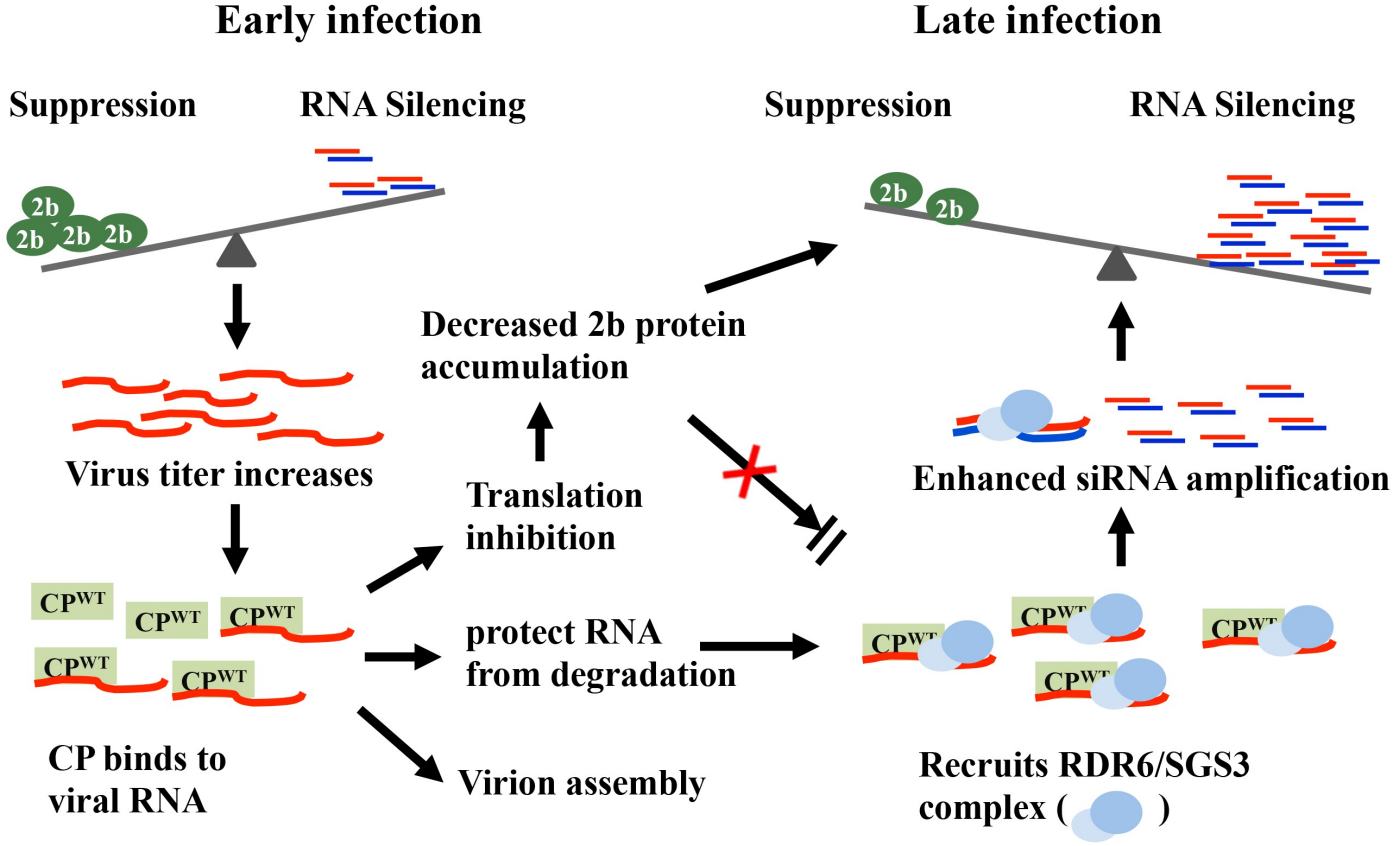
A model explaining the CMV CP-modulated conflict between RNA silencing and 2b-induced suppression in the shoot apices infected with CMV^WT^. During the early infection stages of CMV^WT^, levels of CP^WT^ accumulation are not sufficient to affect accumulation of 2b protein and antiviral silencing. However, at intermediate stages of replication, higher CP^WT^ levels accumulate and bind to viral RNAs to result in virion assembly, reduced 2b protein accumulation and protection of aberrant RNAs from degradation. The CP^WT^ and bounded RNA complex are postulated to recruit RDR/SGS3 for amplification of vsiRNAs that increase antiviral RNA silencing and antagonize 2b protein suppression of host RNA silencing. In summary, CP^WT^ RNA binding inhibits the translation and accumulation of the 2b protein to favor RNA silencing that contributes to viral self-attenuation and long-term symptom recovery.

As the result of our comparative analyses of CP^WT^ and CP^RA^ in the virus infection and GFP co-infiltration assays, we propose the schematic model shown in Fig 8. At early stages of infection, a low level of CP fails to efficiently inhibit 2b protein accumulation or to induce siRNA amplification, which facilitates high-speed replication of viral RNA. Later in infection, abundant CP binds to viral RNA to initiate virion packaging, inhibit RNA translation and facilitate ribonucleoprotein formation for viral movement, as is consistent with the CP functions of other plant viruses, such as BMV and PVA [44, 46]. Simultaneously, saturated CP results in decreased accumulation of 2b protein, which interferes with inhibition of host antiviral RNA silencing. In our experiments, the CP bound RNAs failed to participate in translation and the mRNAs likely were recognized as aberrant RNAs by the RDR/SGS3 complex, and used as substrates for siRNA amplification. Accordingly, the high amounts of CP found at late infection stages reduces synthesis and accumulation of the 2b protein and/or induces siRNA amplification to culminate viral clearance from the shoot apices of infected plants (Fig 8). By comparison, the much lower affinity of CP^RA^ with viral RNA fail to reduce the accumulation of 2b protein efficiently, or induce siRNA amplification. Therefore, we propose that the VSR activities of 2b facilitate continuous infection of CMV^RA^ in SAM regions. Although, it must be noted here that the proposed model is based only on *N*.*benthamiana* and Arabidopsis as the host plants, but since CMV infects more than 1000 species, our proposed model provides the basis for a variety of other experiments in a diverse assay of host plants.

Since a relative healthy host provides a better environment for multiplication within-host and between-host transmission, many plant viruses down-regulate viral virulence to avoid severe disease in order to promote the survival of their hosts [67]. For instance, the strong suppressor P0 suppressor encoded by poleroviruses does not accumulate to detectable levels because of suboptimal translation initiation, which leads to low suppressor activity and reduced viral pathogenicity [68]. The *Tobacco mosaic virus* movement protein also regulates the spread of RNA silencing to self-control viral propagation [69]. Here, our study has revealed a novel self-attenuation mechanism in which the suppression effects of the 2b protein are down-regulated by saturated CP.

Collectively, the roles of the interactions between RNA silencing and virus-encoded suppressors in the co-evolution of hosts and pathogens have been extensively investigated. Our results demonstrate that the CMV CP serves as a central hub to facilitate regulation of dynamically integrated connections between antiviral silencing and VSR activities.

## Materials and methods

### Plant materials and virus inoculation

*N*. *benthamiana* plants were grown in a growth room with a controlled environmental climate programmed for 16 hours (hrs) of light at 24°C and 8 hrs in the dark at 21°C. Seedlings with six to eight fully expanded leaves were used for virus inoculations. For Agrobacterium tumefactions constructions, the cDNAs of RNA1, RNA2, and RNA3, as well as RNA2-Δ2b and RNA3-CP^RA^ were amplified, digested with *Stu* I and *Bam*H I, introduced into pCass4-Rz, and transformed into *A*. *tumefaciens* strain EHA105 [70]. Equal amount of agrobacteria harboring CMV plasmid derivatives were mixed and infiltrated into *N*. *benthamiana* leaves as described previously [71]. All the experiments were repeated at least three times with reproducible results. *Arabidopsis thaliana rdr6-15* (SAIL_617_H07) and *sgs3-1* in Columbia (Col) ecotype were described previously [6]. After vernalized in the dark at 4°C, the seeds were transferred into a growth room with the condition of 10 hrs in light and 14 hrs in dark at 22°C. CMV^WT^ and CMV^RA^ virions propagated in *N*. *benthamiana* leaves were purified and used as a inocula at 100 μg/mL.

### *In situ* hybridization

Shoot and floral apices of infected plants were collected from infected *N*. *benthamiana*, embedded in wax, sectioned, and *in situ* hybridized as described previously [14]. CMV RNA was detected with the digoxigenin (Roche Diagnostics GmbH) labelled vitro-transcribed RNA fragment corresponding to 3′ terminal 200 nucleotides of CMV RNA3, and then detected with antibody anti-digoxigenin conjugated to alkaline phosphatase (Roche Diagnostics GmbH) with nitroblue tetrazolium (NBT) and 5-Bromo-4-Chloro-3-Indolyl Phosphate (BCIP, Sigma). Stained samples were examined with a bright-field microscope (DP72, Olympus) for visualization and photography.

### Agro-infiltration and local suppression of GFP silencing in patch assays

For transient protein expression in *N*. *benthamiana* leaves, CMV CP^WT^, CP^RA^ and 2b cDNAs were introduced into the pGD binary vector [72]. Leaves of 4-week-old *N*. *benthamiana* plants were co-infiltrated with mixed Agrobacterium cultures harboring the positive sense GFP (sGFP) expression plasmid with different combinations of empty vector (V), 2b, and CP plasmids. At 5 dpi, GFP fluorescence in infiltrated leaves was recorded under a long wavelength UV lamp (UVP, California, USA) using a 600D Cannon digital camera [62]. Local suppression assays were independently performed at least three times with reproducible results.

### RNA analysis by Northern blotting and RT-PCR

The topmost infected leaves of 10 to 15 plants were pooled for RNA extraction with Trizol reagent according to the manufacturer instruction (Invitrogen, USA). As described previously [6], 5 μg and 10 μg total RNAs were used for detection of viral RNA and vsiRNAs, respectively. CMV genomic and sgRNAs cDNA detection probes from the 3′ terminal 240 nt of Fny-CMV RNA2 was randomly labeled with [α-^32^P] dCTP. VsiRNAs were detected by the labeled DNA oligonucleotides corresponding to CMV gRNA3 as described previously [6]. The upper uninoculated leaves inoculated with CMV^WT^-Δ2b or CMV^RA^-Δ2b were collected for RNA extraction and detection at 7 dpi. Total RNA was treated with RNase free-DNase I, and used as a template for first-strand cDNA synthesis with M-MLV reverse transcriptase (Promega, USA) as described previously [73]. Viral infections were monitored by RT-PCR using primers corresponding to the RNA3 CP region. Protein phosphatase 2A (PP2A) was used as an RT-PCR control. For local silencing suppression assay, 5 μg and 10 μg total RNA from infiltrated leaves were used for GFP mRNA and siRNA detections, respectively. The randomly-labeled cDNA probe corresponding to GF region (nt 1–400) of GFP cDNA was used for GFP mRNA detection. The GFP-derived siRNA was detected by [α-^32^P] UTP-labeled RNA probe corresponding to GF region of GFP.

### Western blotting analysis

Total proteins extracted from viral inoculated or agro-infiltrated leaf tissue with SDS buffer [100 mM Tris (pH 6.8), 20% glycerol, 4% SDS, and 0.2% bromophenol blue, 10% β-mercaptoethanol] were separated in SDS-PAGE gels, and transferred onto nitrocellulose membranes. Anti-GFP (1:1000), -CP (1:4000), and -2b (1:2000) polyclonal antibodies were used to detect accumulation of GFP, CP, and 2b, respectively. Then, goat anti-rabbit IgG horseradish peroxidase conjugate at a 1:3000 diluted was applied as the secondary antibody, and the membranes were incubated with Pierce ECL Plus chemiluminescent substrate before exposure to x-ray films.

### Protein expression and RNA binding assay

Firstly, amplified CP^WT^ and CP^RA^ cDNAs were introduced into pGEX-KG vectors respectively, and the recombinant plasmids were transformed into BL21. After induction with 0.2mM isopropyl β-D-thiogalactoside(IPTG) at 18°C for 18 h, the resulting GST-tagged fusion proteins and GST tags were purified over Glutathione Sepharose 4B (GE Healthcare) affinity columns according to the manufacturer’s instructions. The RNA binding assays were performed via a described North-Western blot procedure as described [74]. Briefly, 5 μg of purified GST, GST-CP^WT^, or GST-CP^RA^ were separated by 12.5% SDS-PAGE and transferred to nitrocellulose membranes. The membranes were incubated with renaturation buffer (50 mM Tris-HCl, pH 7.5, 0.1% TritonX-100, 10% glycerol, 0.1 mM ZnCl_2_ and 250 mM KCl) overnight at 4°C. Then, the membranes were transferred into binding buffer (10 mM Tris–HCl, pH 7.5, 1 mM EDTA, 100 mM NaCl, 0.05% Triton X-100, and 1X Denhardt’s reagent) containing digoxigenin-11-UTP-labelled (Roche) RNA probe corresponding to CMV RNA 4 or Luc RNA. The bound RNA was blotted with the anti-digoxigen conjugated alkaline phosphatase (1:3000 dilution, Roche) in a NBT/BCIP solution.

### *In vitro* translation assays

The *in vitro* translation assay were performed as described previously [75]. First, the full-length luciferase (Luc) cDNA was introduced into the pMD19-T vector. Then, the bacteriophage T7 promoter and a poly(A) tail were inserted at the Luc cDNA N- and C- termini, respectively, and the resulting *Xba* I-linearized plasmid was used as a template for *in vitro* transcription by the mMESSAGE T7 kit (Ambion, USA). Two micrograms (μg) of Luc mRNA and different concentrations of purified GST-CP^WT^, GST-CP^RA^ or GST were translated in the Wheat Germ Extract Plus kit (Promega, USA) for 2 hours at 25°C. Then, luciferase activity of translated products was determined with a 20/20 luminometer (Promega, USA) as described previously [76].

### *In vivo* coimmunoprecipition assays

Coimmunoprecipition of CMV CP and AtSGS3 was performed as described previously [77]. *N*. *benthamiana* plants were agroinfiltrated for transient expression of Flag-CP^WT^, Flag-CP^RA^ with GFP-SGS3 or GFP and leaf tissues were homogenized in coimmunoprecipition buffer [10% glycerol, 25 mM Tris-HCl (PH7.5), 200 mM NaCl, 1 mM EDTA, 0.1% Tritonx-100, 2% PVP-40, 50 μM MG132, 10 mM DTT and cocktail]. After filtration and centrifugation, the supernatants were incubated with anti-Flag M2 affinity gel (Sigma) for 2 hrs followed by washing five times. The immunoprecipitated products were boiled with SDS buffer for Western blotting assays with corresponding antibodies.

### Bimolecular fluorescence complementation (BiFC) assays

BiFC assays were performed with minor modifications as described previously [78]. AtSGS3 and CP^WT/RA^ cDNA fragments, and were the RuBisco control protein (Rub) were cloned into the BiFC vectors pSPYNE-35S and pSPYCE-35S, respectively. *A*. *tumefaciens* EHA105 strains containing the recombinant BiFC plasmids and the tomato bushy stunt virus P19 plasmid were co-infiltrated into *N*. *benthamiana* leaves at a final ratio of 0.5:0.5:0.3 (OD_600_) and epidermal cells of infiltrated leaves were observed for fluorescence analysis (YFP) at 2 dpi using confocal laser scanning microscopy (CLSM) (Olympus FV1000).

## Supporting information

S1 Fig An alanine substitution in the N terminus of the R-rich CP motif reduced the RNA-binding capability of CP, but not formation of viral particles during infection. (A) Electron micrographs depicting viral particles purified from leaves systemically infected with CMV^WT^ or CMV^RA^ at 7 dpi. Bars = 100 nm. (B) Detection of protected viral RNAs in virions (10 μg) that underwent an incubation with RNase A (0.02 μg/μl) for 0, 15 and 30 min, followed by RNA extractions for Northern blotting. Note: CMV^WT^ protected viral RNA from substantial degradation for 15 min, whereas CMV^RA^ viral RNA was completely degraded after 15 min.
(TIF)

S2 Fig Requirement of the 2b protein for systemic infection of *N. benthamiana* plants with CMV^WT^ and CMV^RA^. (A) Symptoms of *N*. *benthamiana* plants infected by CMV ^WT^-Δ2b (middle) and CMV^RA^-Δ2b (right). Infected plants were photographed at 7 dpi. (B) Western blotting showing CMV CP expression in infiltrated leaves and (C) upper uninoculated leaves of three independent experiments with the antiserum of CMV CP. Mock-infected plants were used as the negative control. Coomassie brilliant blue (CBB) staining was used as a protein loading controls, and three independent experiments with similar controls were conducted. (D) Viral RNA accumulation in upper non-inoculated leaves was detected by RT-PCR with primers that annealed to the RNA3 CP region. The phosphatase 2A (PP2A) was used as a positive RT-PCR control.
(TIF)

S3 Fig CMV^WT^ induced more potent antiviral silencing than CMV^RA^ in emerging *Arabidopsis thaliana* leaves. (A) CMV^RA^ caused severe symptoms in the newly grown tissues of *A*. *thaliana* plants (middle), whereas CMV^WT^ only induced mosaic symptoms in the full expanded leaves of *A*. *thaliana* plants (right). Infected plants were photographed at 21 dpi. CMV^RA^ induced extensively curled leaves in the newly emerging tissues, and resulted in late bolting and reductions in apical dominance at 35 and 56 dpi, respectively. (B) Accumulation of viral genomic/subgenomic RNAs (C) and vsiRNAs derived from viral genomic RNA3 in emerging leaves of *A*. *thaliana* plants infected with CMV^RA^ and CMV^WT^ at 21 dpi. The 25S rRNA and U6 RNAs were used as loading controls for the high and low molecular weight RNAs, respectively.
(TIF)

S4 Fig Conserved cucumovirus N terminal R-rich CP regions and their roles in 2b-mediated suppression of RNA silencing in the transient expression systems. (A) Alignment of N terminal coat protein sequences from different CMV subgroups and tomato aspermy virus (TAV). The conserved R-rich region is indicated. (B) GFP fluorescence in *N*. *benthamiana* leaves after agroinfiltration of sGFP reporter constructs (OD_600_ = 0.4) in combination with the pGD empty vector (V, OD_600_ = 0.4), pGD-derived vector encoding 2b (OD_600_ = 0.2) and different Myc-tagged CP proteins (OD_600_ = 0.4), as indicated. Photographs were taken under UV light at 5 dpi. (C) Western blotting analysis of samples extracted from infiltrated regions shown in panel B. Anti-GFP, -Myc, and -2b polyclonal antibodies were used to detect the accumulation of the GFP, CP, and 2b proteins, respectively. (D) GFP fluorescence in local leaves of *N*. *benthamiana* with agroinfiltration of reporter sGFP, combined with pGD empty vector, Myc-tagged TAV 2b protein and Myc-tagged TAV CP as indicated in the panel. (E) Western blotting analysis with samples extracted from infiltrated regions of panel D and analyzed as described in Panel C. Coomassie brilliant blue (CBB) staining was used as the protein loading control.
(TIF)

S5 Fig CMV CP attenuatation of VSRs-mediated suppression of local GFP silencing during transient expression. (A) GFP fluorescence in local leaves of *N*. *benthamiana* after agroinfiltration of reporter sGFP vectors (OD_600_ = 0.4), combined with pGD empty vector (V, OD_600_ = 0.4), Flag-tagged VSRs (OD_600_ = 0.2) and CPs (OD_600_ = 0.4), as indicated. Photographs were taken under UV light at 5 dpi. VSR proteins include P19, HC-Pro, and P38 from *Tomato bushy stunt virus* (TBSV), *Tobacco etch virus* (TEV), and *Turnip crinkle virus* (TCV), respectively. (B) Western blots analysis with samples extracted from agroinfiltrated regions of leaves shown in panel A. Anti-GFP, -CP, and -Flag polyclonal antibodies were used to detect the accumulation of GFP, CP, and VSRs, respectively. (C) GFP fluorescence (middle panel) in regions agroinfiltrated with the sGFP reporter vector (OD_600_ = 0.4), together with P19 (OD_600_ = 0.2) and different CP concentrations (OD_600_ = 0–0.8), as indicated in left panel. Western blotting analyses of samples extracted from infiltrated region is shown in the right panel. Anti-GFP and anti-CP polyclonal antibodies were used to detect accumulation of GFP and CP, respectively. Mock-infected plants were used as a negative control. The coomassie brilliant blue (CBB) staining were used as protein loading controls.
(TIF)

S6 Fig Failure of CP^WT^ and CP^RA^ to interact with SGS3 in Yeast-two-hybrid assay. (A) Full-length cDNAs of SGS3 and Impα1 clones was fused to the GAL4 DNA binding domain in pGBKT7 vector, and CP^WT^ and CP^RA^ cloned cDNAs were fused to the GAL4 activation domain of the pGADT7 vector, respectively. Combination of plasmids were co-transformed into the yeast stain AH109 (Left panel). All transformants were grown at 30°C on media lacking Trp and Leu, and then transferred to media lacking Trp, Leu, His and Ade. (B) Western blotting analyses were performed to determine expression of the SGS3 and CPs proteins.
(TIF)
