# Supplementary figures and images for: Cucumber mosaic virus coat protein modulates the accumulation of 2b protein and antiviral silencing that causes symptom recovery in planta
Source: PLoS Pathog. 2017 Jul 20;13(7):e1006522. doi: 10.1371/journal.ppat.1006522 (PMC5538744; doi:10.1371/journal.ppat.1006522)

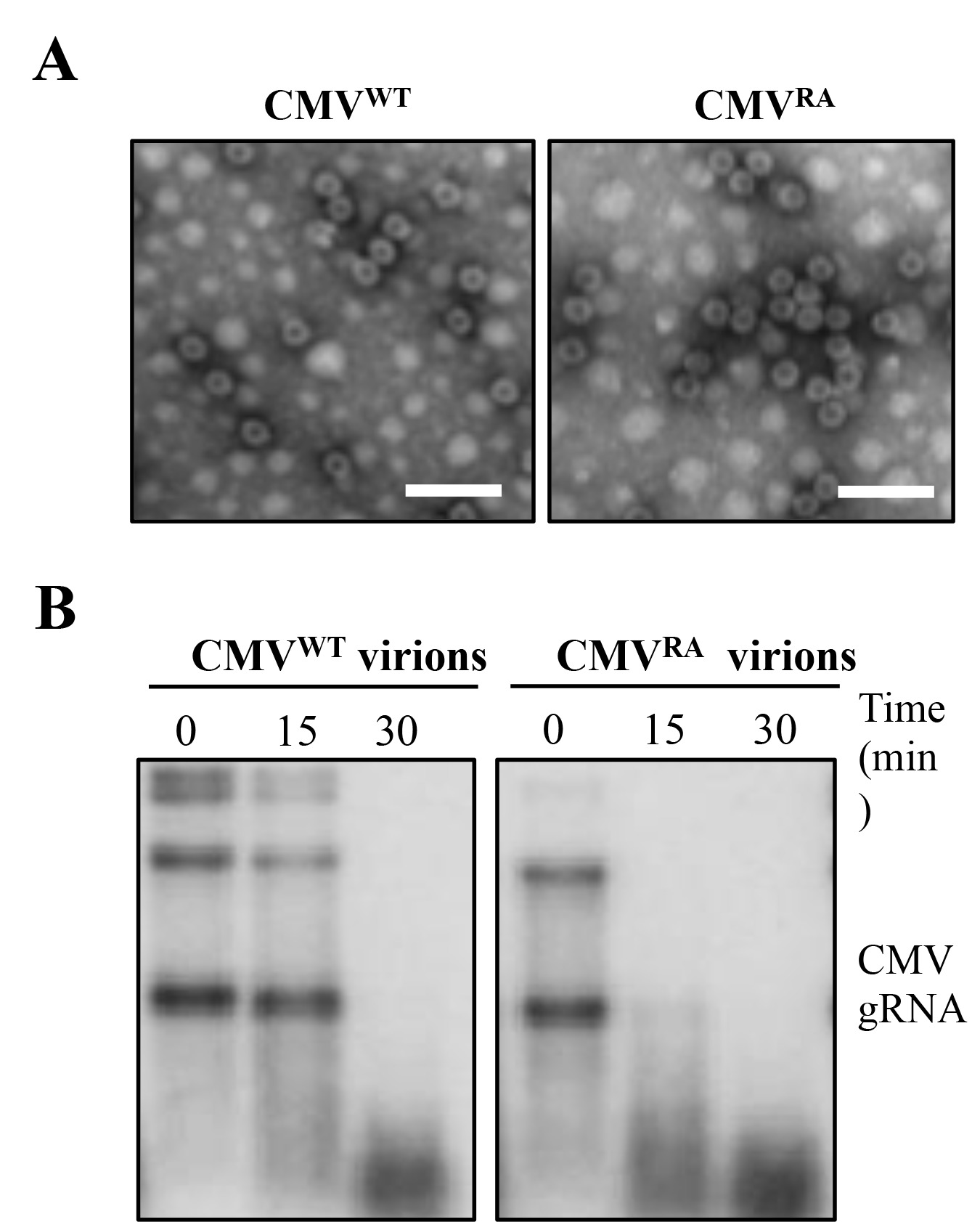

Supplement: S1 Fig — (A) Electron micrographs depicting viral particles purified from leaves systemically infected with CMVWT or CMVRA at 7 dpi. Bars = 100 nm. (B) Detection of protected viral RNAs in virions (10 μg) that underwent an incubation with RNase A (0.02 μg/μl) for 0, 15 and 30 min, followed by RNA extractions for Northern blotting. Note: CMVWT protected viral RNA from substantial degradation for 15 min, whereas CMVRA viral RNA was completely degraded after 15 min. (TIF) [file ppat.1006522.s001.tif]

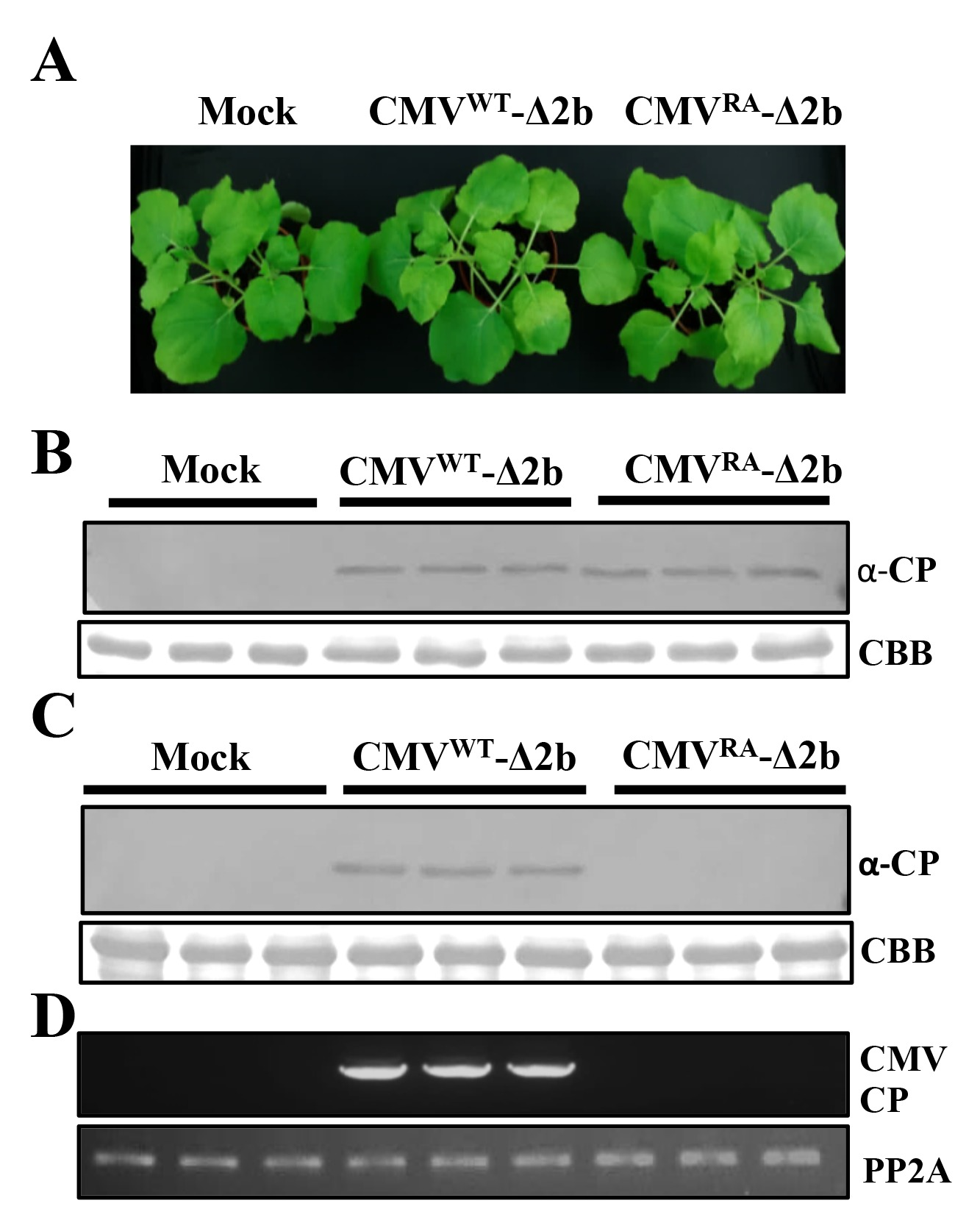

Supplement: S2 Fig — (A) Symptoms of N. benthamiana plants infected by CMV WT-Δ2b (middle) and CMVRA-Δ2b (right). Infected plants were photographed at 7 dpi. (B) Western blotting showing CMV CP expression in infiltrated leaves and (C) upper uninoculated leaves of three independent experiments with the antiserum of CMV CP. Mock-infected plants were used as the negative control. Coomassie brilliant blue (CBB) staining was used as a protein loading controls, and three independent experiments with similar controls were conducted. (D) Viral RNA accumulation in upper non-inoculated leaves was detected by RT-PCR with primers that annealed to the RNA3 CP region. The phosphatase 2A (PP2A) was used as a positive RT-PCR control. (TIF) [file ppat.1006522.s002.tif]

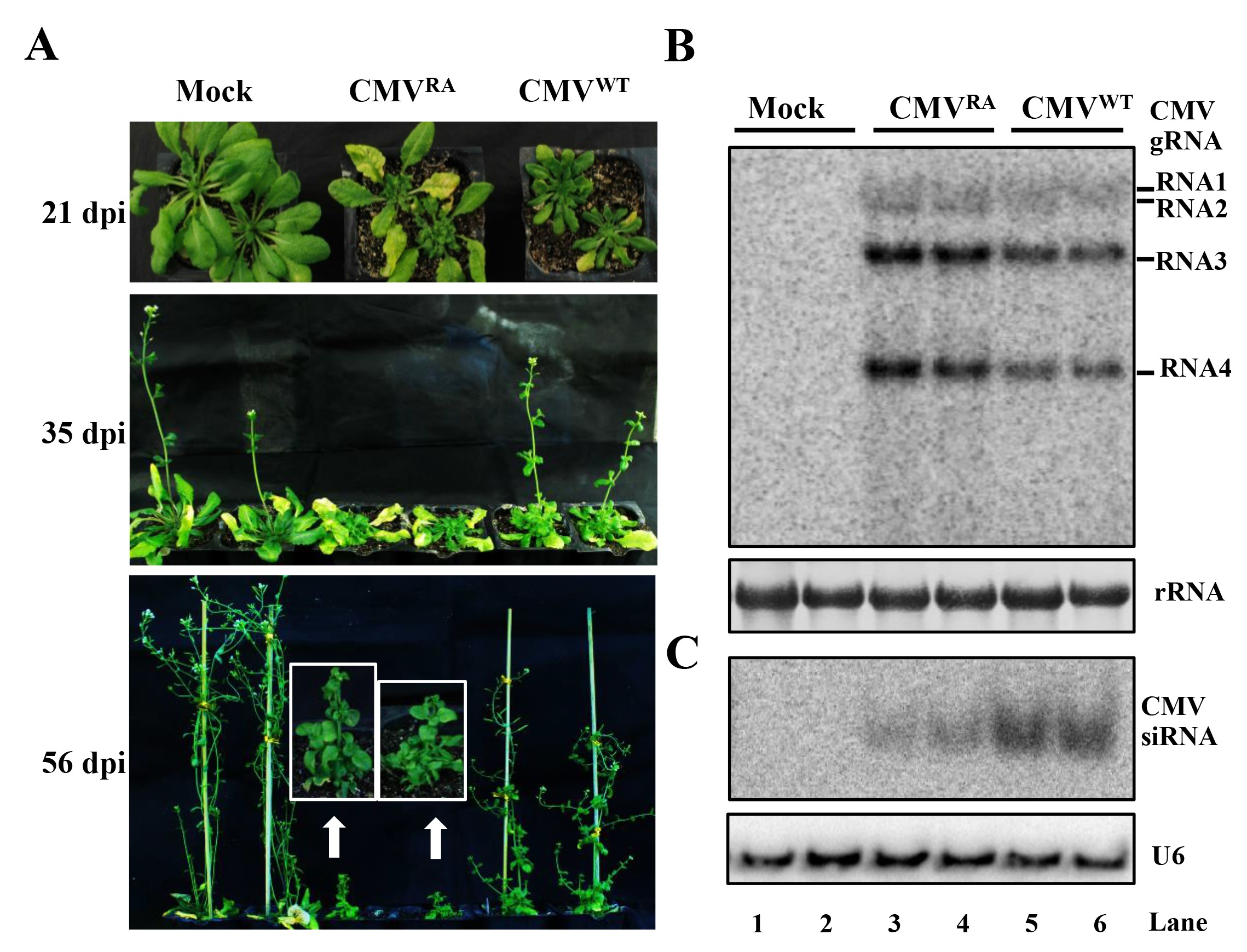

Supplement: S3 Fig — (A) CMVRA caused severe symptoms in the newly grown tissues of A. thaliana plants (middle), whereas CMVWT only induced mosaic symptoms in the full expanded leaves of A. thaliana plants (right). Infected plants were photographed at 21 dpi. CMVRA induced extensively curled leaves in the newly emerging tissues, and resulted in late bolting and reductions in apical dominance at 35 and 56 dpi, respectively. (B) Accumulation of viral genomic/subgenomic RNAs (C) and vsiRNAs derived from viral genomic RNA3 in emerging leaves of A. thaliana plants infected with CMVRA and CMVWT at 21 dpi. The 25S rRNA and U6 RNAs were used as loading controls for the high and low molecular weight RNAs, respectively. (TIF) [file ppat.1006522.s003.tif]

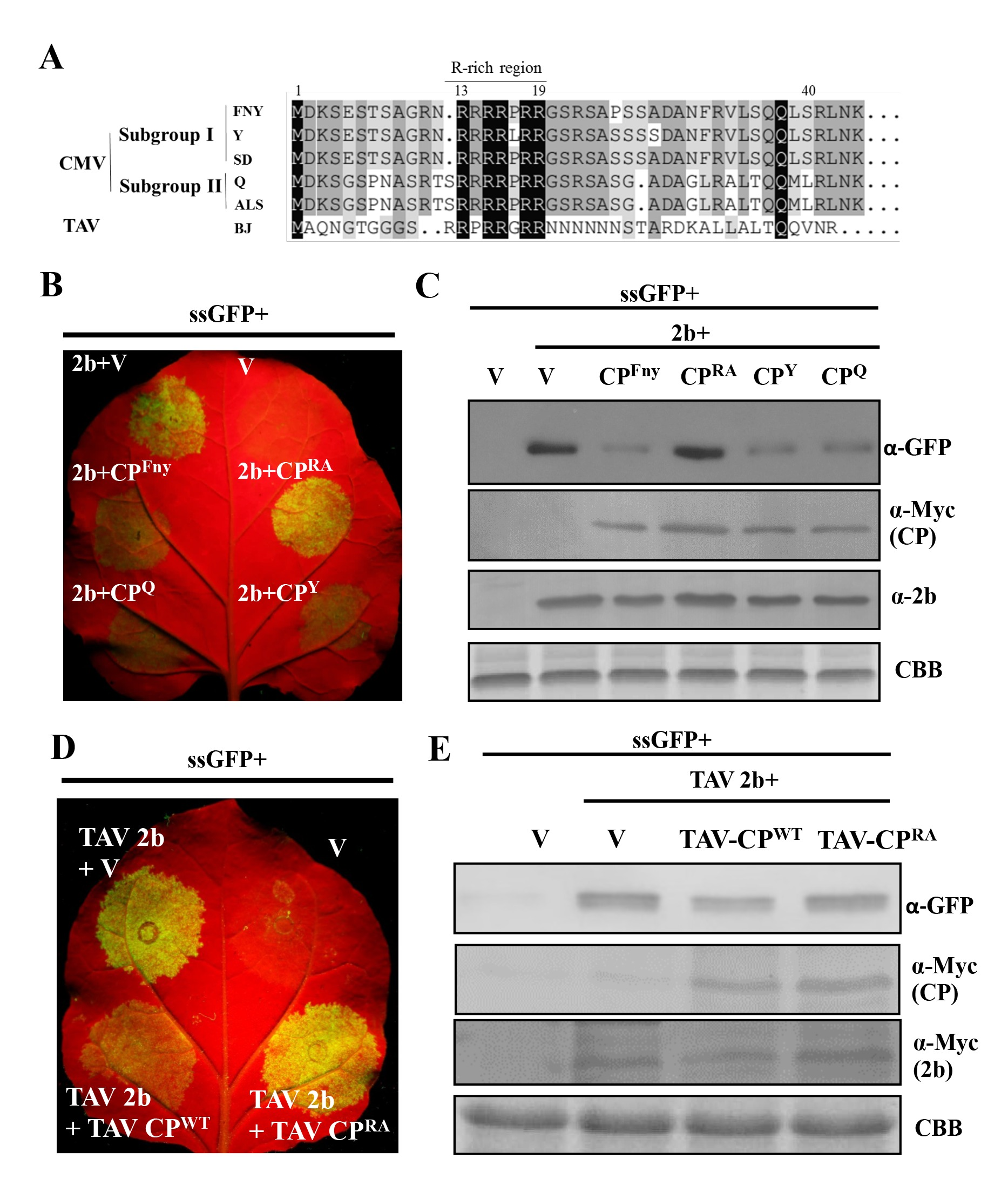

Supplement: S4 Fig — (A) Alignment of N terminal coat protein sequences from different CMV subgroups and tomato aspermy virus (TAV). The conserved R-rich region is indicated. (B) GFP fluorescence in N. benthamiana leaves after agroinfiltration of sGFP reporter constructs (OD600 = 0.4) in combination with the pGD empty vector (V, OD600 = 0.4), pGD-derived vector encoding 2b (OD600 = 0.2) and different Myc-tagged CP proteins (OD600 = 0.4), as indicated. Photographs were taken under UV light at 5 dpi. (C) Western blotting analysis of samples extracted from infiltrated regions shown in panel B. Anti-GFP, -Myc, and -2b polyclonal antibodies were used to detect the accumulation of the GFP, CP, and 2b proteins, respectively. (D) GFP fluorescence in local leaves of N. benthamiana with agroinfiltration of reporter sGFP, combined with pGD empty vector, Myc-tagged TAV 2b protein and Myc-tagged TAV CP as indicated in the panel. (E) Western blotting analysis with samples extracted from infiltrated regions of panel D and analyzed as described in Panel C. Coomassie brilliant blue (CBB) staining was used as the protein loading control. (TIF) [file ppat.1006522.s004.tif]

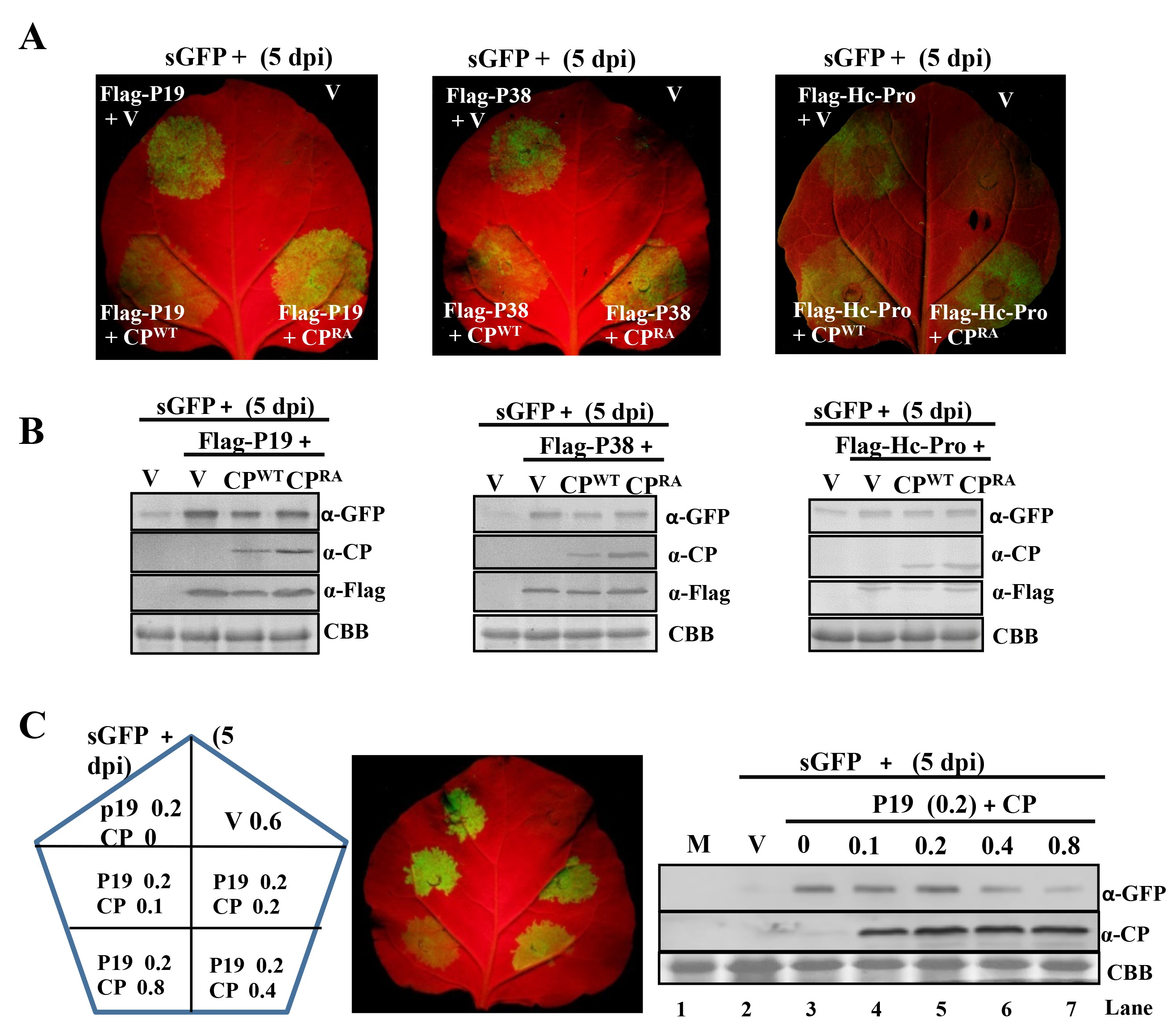

Supplement: S5 Fig — (A) GFP fluorescence in local leaves of N. benthamiana after agroinfiltration of reporter sGFP vectors (OD600 = 0.4), combined with pGD empty vector (V, OD600 = 0.4), Flag-tagged VSRs (OD600 = 0.2) and CPs (OD600 = 0.4), as indicated. Photographs were taken under UV light at 5 dpi. VSR proteins include P19, HC-Pro, and P38 from Tomato bushy stunt virus (TBSV), Tobacco etch virus (TEV), and Turnip crinkle virus (TCV), respectively. (B) Western blots analysis with samples extracted from agroinfiltrated regions of leaves shown in panel A. Anti-GFP, -CP, and -Flag polyclonal antibodies were used to detect the accumulation of GFP, CP, and VSRs, respectively. (C) GFP fluorescence (middle panel) in regions agroinfiltrated with the sGFP reporter vector (OD600 = 0.4), together with P19 (OD600 = 0.2) and different CP concentrations (OD600 = 0–0.8), as indicated in left panel. Western blotting analyses of samples extracted from infiltrated region is shown in the right panel. Anti-GFP and anti-CP polyclonal antibodies were used to detect accumulation of GFP and CP, respectively. Mock-infected plants were used as a negative control. The coomassie brilliant blue (CBB) staining were used as protein loading controls. (TIF) [file ppat.1006522.s005.tif]

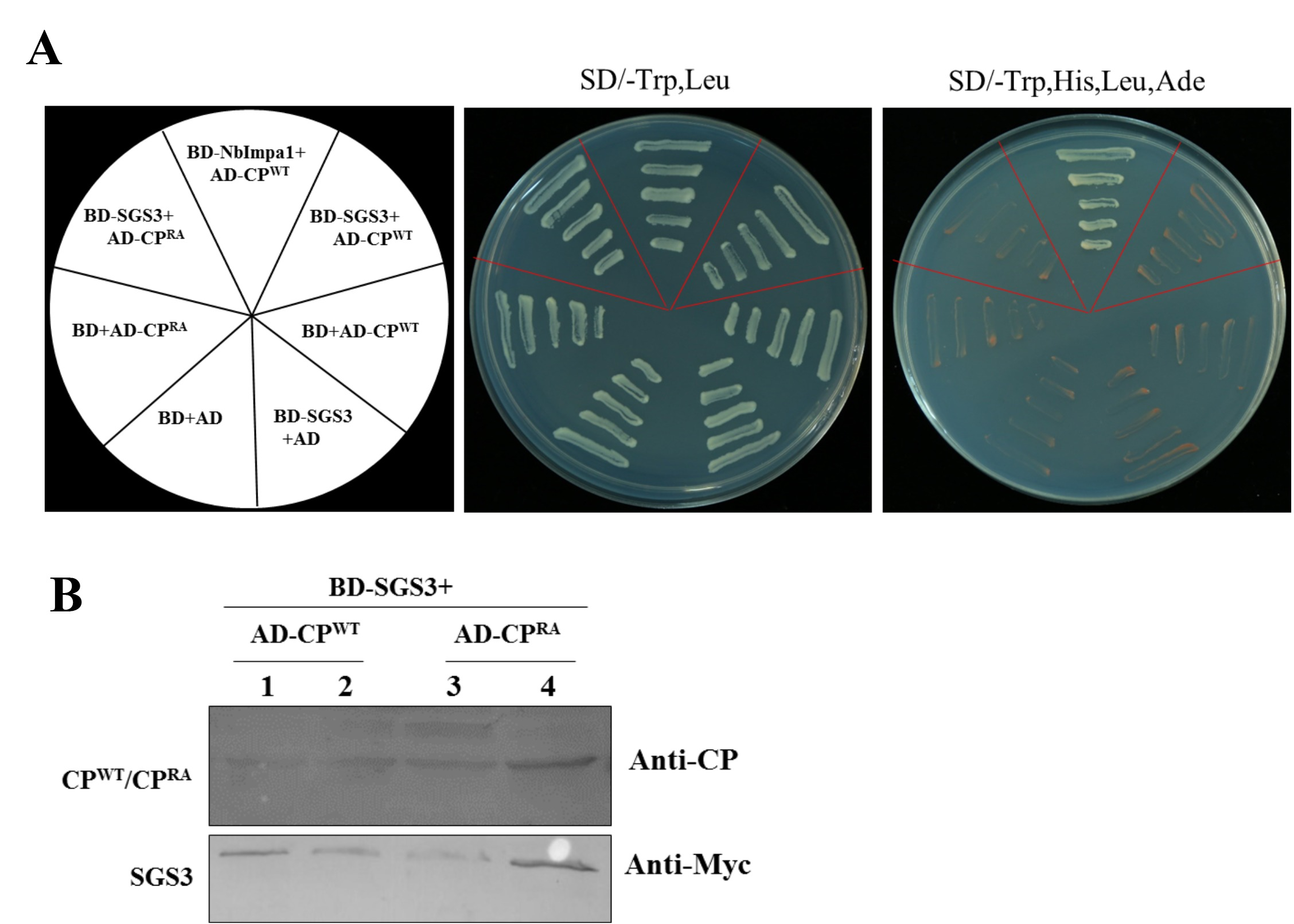

Supplement: S6 Fig — (A) Full-length cDNAs of SGS3 and Impα1 clones was fused to the GAL4 DNA binding domain in pGBKT7 vector, and CPWT and CPRA cloned cDNAs were fused to the GAL4 activation domain of the pGADT7 vector, respectively. Combination of plasmids were co-transformed into the yeast stain AH109 (Left panel). All transformants were grown at 30°C on media lacking Trp and Leu, and then transferred to media lacking Trp, Leu, His and Ade. (B) Western blotting analyses were performed to determine expression of the SGS3 and CPs proteins. (TIF) [file ppat.1006522.s006.tif]
